# Development and Assessment of a Novel Generic Finite Element Spine Model for Clinical Applications

**DOI:** 10.1002/cnm.70098

**Published:** 2025-09-22

**Authors:** Yifan Su, Athanasios I. Tsirikos, Vasileios Koutsos, Pankaj Pankaj

**Affiliations:** ^1^ Institute for Bioengineering, School of Engineering The University of Edinburgh Edinburgh UK; ^2^ Scottish National Spine Deformity Centre – Royal Hospital for Children and Young People Edinburgh UK; ^3^ Institute for Materials and Processes, School of Engineering The University of Edinburgh Edinburgh UK

**Keywords:** facet joint forces, finite element analysis, generic model, intradiscal pressure, range of motion, spine biomechanics

## Abstract

Numerical modeling has been extensively employed to understand the biomechanics of the spine. Often, patient‐specific models developed from medical scans, which are specific to an individual and their particular clinical case, are used. The aim of this study was to develop a generic model of the full adolescent spine, which includes ribs, muscles, and ligaments, that can effectively simulate realistic spinal biomechanics. The model was developed using computer‐aided design, incorporating anatomical parameters to represent a 15‐year‐old adolescent full‐spine geometry. Essential components like the ribcage and related musculature were included to capture realistic biomechanics. The model appraisal involved mesh sensitivity analysis and tests on selected functional spinal units (FSUs) in each spinal region to assess the biomechanics of specific components of the full spine. Biomechanical responses, including range of motion, intradiscal pressure, and facet joint forces, were evaluated across multiple simulated loading tasks. Results were compared to previous in vitro and in silico studies. Our model demonstrated good agreement with previous experimental and numerical studies. The ribcage inclusion simulated the stiffening effect observed in vivo satisfactorily. Ligamentous effect tests on thoracic and lumbar FSUs indicated that the model satisfactorily replicated expected biomechanical responses. The study shows that the developed model can be employed effectively to simulate real‐life spine motions. The developed model will be used for future AIS research, enabling the investigation of surgical treatment outcomes across diverse clinical scenarios.

## Graphical abstract

This study proposes and develops a generic finite element model of the adolescent spine, designed to replicate realistic biomechanics for clinical applications. Incorporating ribcage and musculature, the model captures key biomechanical behavior, including spinal motion and loading. Assessment against previous experimental and computational data confirms its accuracy in predicting range of motion, intradiscal pressure, and facet joint forces.

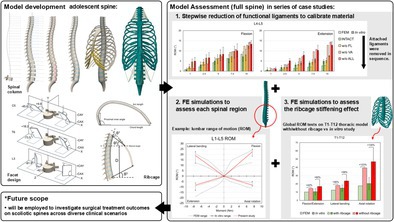

## 1 Introduction

Biomechanics of the human spine has been mostly studied using either in vitro or in silico approaches. As the in vitro approach was commonly adopted, most in silico models were developed and validated using in vitro studies and contributed in their attempt to replicate the in vivo reality for clinical applications.

In vitro studies conducted using cadaveric spine specimens have been valuable in understanding the spine anatomy [1] and fundamental biomechanical functions of the spinal column [2]. The biomechanical responses measured in vitro (range of motion [3], intradiscal pressure, facet joint forces [4], etc.) have been used to describe the mobility and stability of the spine [5] in translations, angulations, and under anticipated loads. Consequently, in silico models employ the boundary and loading conditions from the in vitro test setups and aim to achieve in vivo‐like load–displacement curves in different body postures and a satisfactory load bearing capacity within the in vitro corridors.

There has been a rising number of in silico spine models employed for biomechanical evaluation of surgical risks and for predicting treatment outcomes using the finite element (FE) models [6, 7, 8, 9]. In patient‐specific models, the geometry is obtained using scans of individual patients' anatomy. These studies rely on material properties from other studies, only apply to the specific patient whose scan was used, and require significant time and resources to conduct. A patient‐specific model, therefore, may not be able to answer clinical questions related to a more general patient population.

Also, most FE studies focus on a single human spine region in normal [10] and abnormal cases [11]. A large number of studies have generated and tested numerical intervertebral disc (IVD) models using fewer spine segments, typically with a single functional spinal unit (FSU) [12, 13]. Thoracolumbar models that involve multiple spine regions have been useful in assessing the global effect of the mechanical properties of spinal components [14, 15] but are not specifically designed to assess or plan spinal surgery outcomes. Thus, instead of relying on patient‐specific data, a few recent FE studies have adopted the parametric modeling approach to develop generic spine geometries to represent specific populations and fulfill particular research purposes.

A parametric generic model based on average parametric data of the spine morphology, which is not subject‐specific, can help elicit answers to a range of common questions of clinical relevance. Grünwald et al. [16] used a healthy adult thoracolumbar spine with ribcage to simulate scoliosis cases. Although the model enabled translations on vertebrae to simulate scoliotic conditions and was effective in assessing the pattern of stress and strain distribution across IVDs, the impact of ligaments and spinal joints was not investigated. Roth et al. [17] analyzed the effect of changing the number of instrumentation levels on a validated healthy thoracolumbar model whose geometry was developed through parametric definitions. However, the impact of ribcage structure and related musculature was not considered in their research. Bellina et al. [18] implemented functions of morphological dependent parameters in computer‐aided design (CAD) software and automatically generated a fully‐parametric thoracolumbar spine with ribcage, but the study did not consider the effect of the muscles on the thoracolumbar spine biomechanics under realistic loading tasks.

The ultimate aim of the in silico studies on spine is to assist the clinical diagnosis and treatments by creating healthy and abnormal geometries and simulating certain in vivo situations and surgical interventions [7, 19]. Computational models should be readily accessible to provide guidance to clinicians. The aim of this study is to develop a generic in silico full spine model with all necessary spine components that contribute to realistic spine biomechanics. The ribcage and its related musculatures are included so that the developed spine can be employed to simulate real‐life spine motions, study spinal diseases, and assess related clinical treatments.

This study discusses the development of the model and assesses it by evaluating the influence of a range of components and subjecting it to a series of load cases. The results are compared with the published in vitro and in silico studies.

## 2 Methods

### 2.1 Parametric Spine Model Design

A generic adolescent full‐spine model was developed using CAD software. The model included 22 vertebrae in total, involving the thoracolumbar spine segments (T1–L5) and two complementary components—the lower cervical region (C3–C7) and sacrum.

**FIGURE 1:**
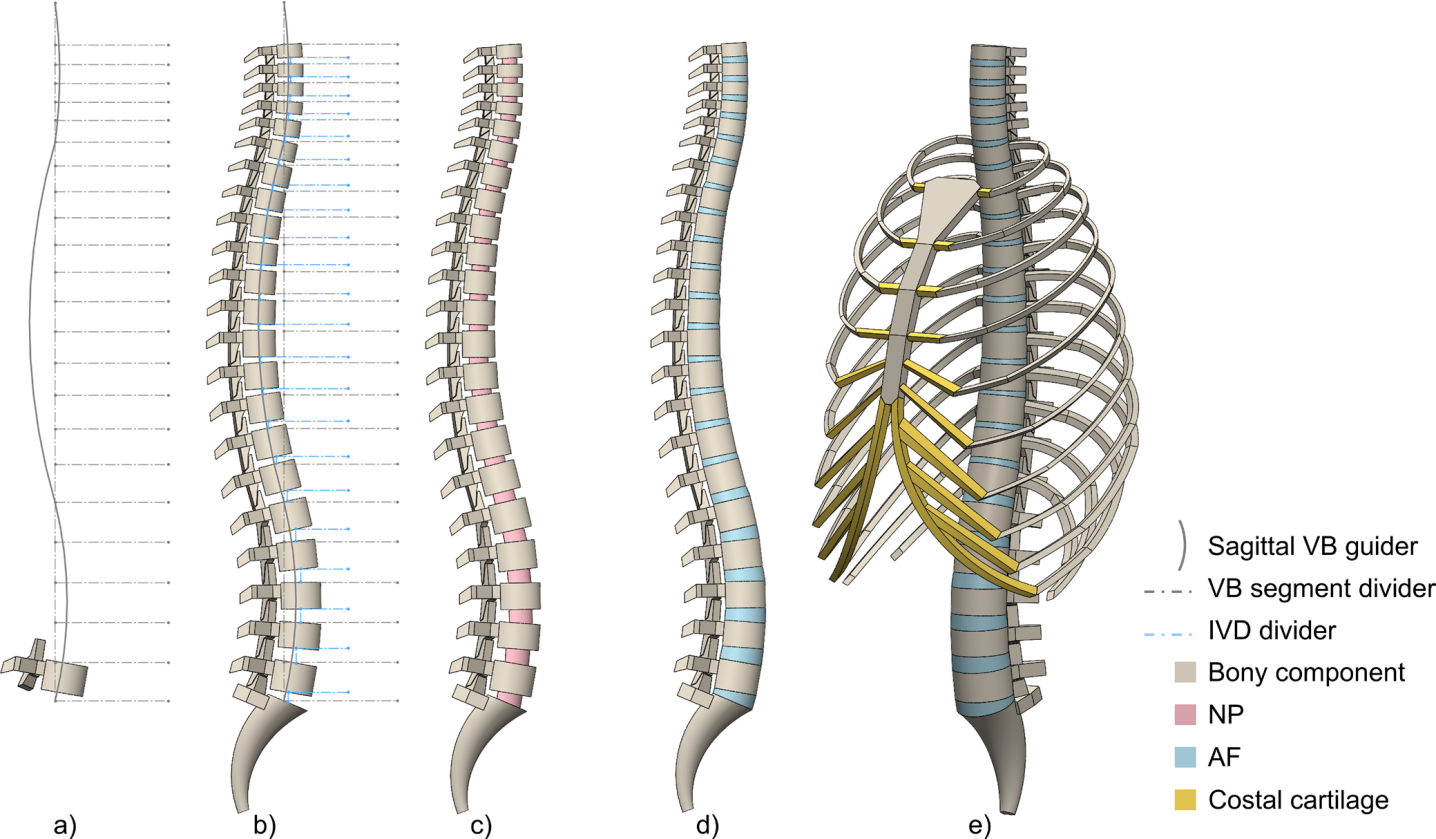
Adolescent spine model development. (a) Grey curves determined the sagittal curvature of the cervical, thoracic and lumbar region, guiding the vertebral sagittal alignment; grey dashed lines marked the sagittal vertical height of each vertebral segment (VB and its following IVD). (b) Blue dashed lines marked the sagittal vertical height of IVDs in each vertebral segment. (c) NPs at each level are shown. (d) AFs at each level are shown. (e) The thoracic cage geometry was assembled with the complete spinal column model, including 12 pairs of ribs, sternum and costal cartilages.

**FIGURE 2:**
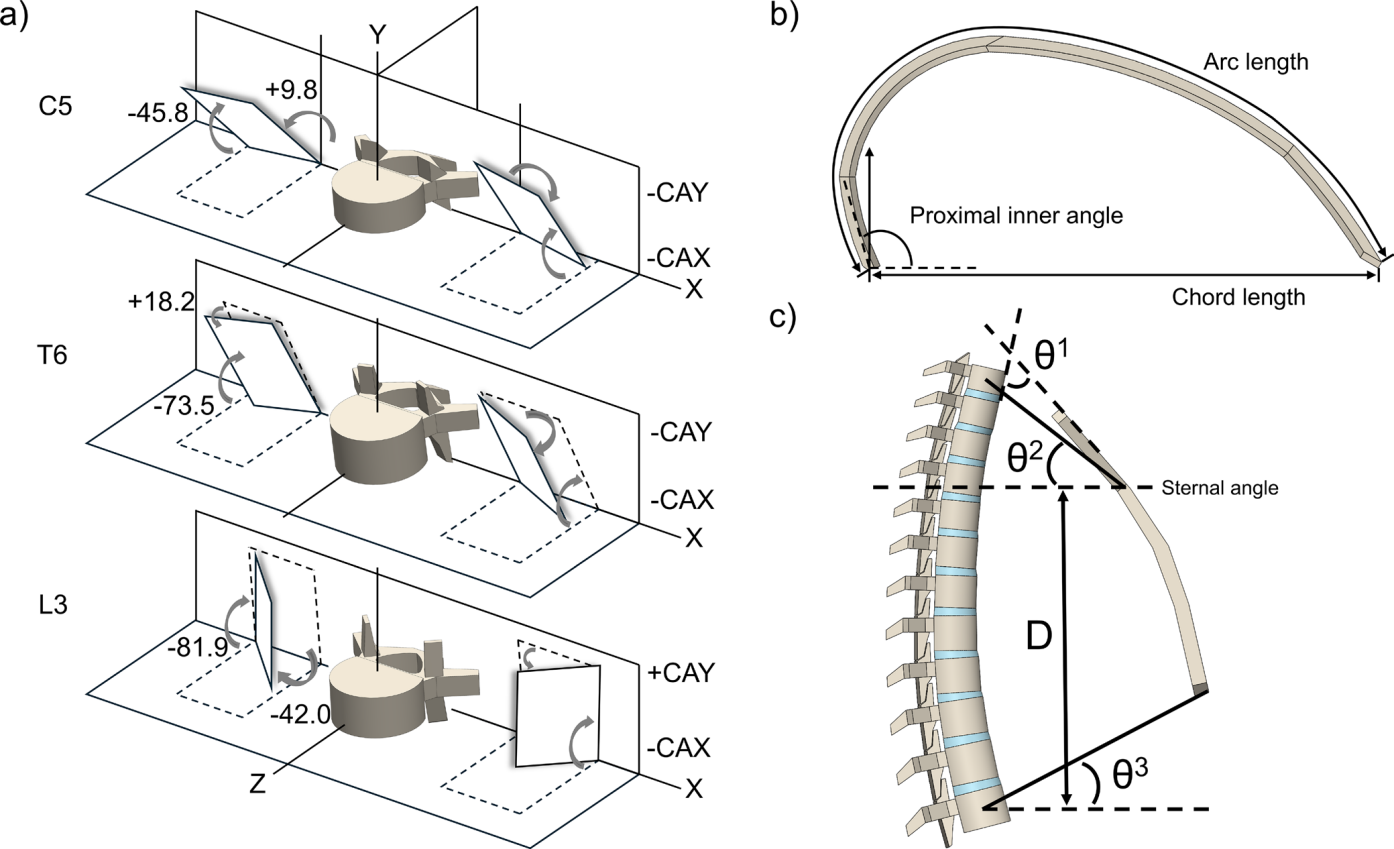
Adolescent spine model design. (a) The FJ design of the generic spine model was differentiated in three spine sections, C5, T6, and L3 vertebrae are shown as examples, the card angle about *X* and *Y* axes (CAX, CAY) of the inclined facet surfaces employed previous findings [24]. (b) The rib morphology was determined by three main shape parameters: Rib chord length [25], arc length [26] and proximal inner angle [18, 25]. (c) The ribcage morphology was determined by several shape parameters [18, 27]: the manubriosternal joint location was marked by the sternal angle, lying at the T4–T5 FJ region; the thoracic and abdominal cavity were configured according to the angle *θ* ^1^ between sternum and T1–T12 anterior wall, the angle *θ* ^2^ between sternum upper edge and T1 centroid, the angle *θ* ^3^ between sternum upper edge and T12 centroid and the distance D from sternum upper edge to T12 centroid.

Each vertebra of the spinal column was created in SOLIDWORKS 2022 (SolidWorks Corporation, Dassault Systèmes, France). The sagittal vertical heights at each spine level were derived from the relative proportions of vertebral segments [20] (Figure 1a). The adolescent vertical spine height was derived based on the normal spine growth [21, 22] of the target age group. The model generation was initiated by defining the sagittal guideline for the vertebral alignment, applying the average adolescent C1–C7 cervical lordosis, T1–T12 thoracic kyphosis, and L1–L5 lumbar lordosis as sagittal parameters. The vertebra model was initially extruded as an elliptic cylinder while its posterior elements (spinal processes, vertebral arches) were connected through rectangular extruded sections. The spinal canal was considered as an elliptic hollow cylinder intersected by vertebral bodies (VBs). The relative positions of the spinal canal and VB were determined by estimating the longitudinal transverse distance between the centres of two intersecting cylinders. The vertebral depth and the spinal canal depth were input to calculate the distance [16]. The ratio of vertebral width to depth was derived from the literature [1, 23], and its variation was assumed to follow the sagittal morphology. Sagittal vertical height separation lines were marked for each VB segment (i.e., vertebra with its following IVD) according to the respective proportion of the full‐spine height occupied (Figure 1b). The C3–L4 vertebrae were assembled following the sagittal guidelines. The facet joints (FJs) were generated by splitting the extruded prisms between adjacent vertebrae to create gaps and form pairs of inclined opposing FJ surfaces (Figure 2). The IVD height of the spinal column model was derived from the documents of paediatric cervical IVD height [28] and dimensions of the complete human spine [29], highlighted as the IVD separation lines shown in Figure 1b. The VB height at each level was then calibrated according to the derived IVD height. The adjacent levels of the spinal column model were lofted together to generate the IVDs, each involving an inner portion nucleus pulposus (NP) which was surrounded by an outer portion annulus fibrosus (AF).

The ribs were defined by proximal inner angles, arc lengths, and chord lengths [25] integrating a constant scaling ratio relative to the adult ribcage geometry, as ribcage growth with time has been shown to be a linear process [30]. Each rib was created using the sweep function and connected to the corresponding VB and posterior part. The sternum consisted of two extruded sections, the sternal manubrium and body, whose position was defined based on the sternal angle [31] and the sagittal parameters of the thoracic cage [27]. The costal cartilage was created using the loft function, connecting the ribs to seven pairs of sternal notches on both sides of the sternum model, whereas the cartilages of the seventh to tenth ribs were joined together, forming the costal margin. A simplified sacrum was lofted between the bottom coccyx and the caudal part (L5 IVD) of the spinal column model for establishing the torso musculature.

#### 2.1.1 Parametric Model Generation

Anatomical parameters were derived from literature to generate a generic model, representing a normal spine of a 15‐year‐old adolescent. The model was not gender specific. The spinal column model height (C3–L5) was 50.9 cm, with a consistent cervical lordosis, thoracic kyphosis, and lumbar lordosis of 14.9° [32], 32.5° [33], and 27.5° [33], respectively. As the IVDs determine the global mobility of the spine [5], the average cervical IVD heights (C3–C7) of the 15‐year‐old group [28] and the average adult IVD heights (C3–L5) [29] were used to derive the IVD heights for the generic model. The VB heights were modified according to the derived IVD height at each level. Each NP had a cross‐sectional area covering approximately 40% of the entire IVD area [34]. The FJs were individually developed for the cervical, thoracic, and lumbar regions due to the anatomical differences in orientations (Figure 2) [24]. Assuming the inter‐facet distances between the opposing facets were constant for all spine levels, a 0.5 mm gap was created for each facet. The facet cartilages were not included at this stage of the model development. The overall ribcage dimension of the 15‐year‐old adolescent spine model was assumed to reach 96.67% of the final size at skeletal maturity [30]. The parameters used to develop the ribcage model, presented by Bellina et al. [18], Burgos et al. [27], and Dansereau et al. [26], are employed in this study, shown as Figure 2b,c. The proximal inner angle was the mean value from a previous ribcage study [25].

### 2.2 FE Spine Model Development

The constructed generic parametric spine model (Figure 1e) was imported into *Abaqus* (Dassault Systèmes, France) to generate a FE model, which included meshing, assigning material properties, and inserting elastic components as ligaments and musculatures.

#### 2.2.1 Mesh and Material Assignment

The full spine model was meshed using 10‐noded quadratic tetrahedral elements (C3D10) except for the facet cartilages. Different parts of the spine were meshed separately, and surfaces connected used tie constraints except for the FJ surface contacts; similar element sizes were used between contact surfaces. The facet cartilages were 0.1 mm offset mesh layers created on each pair of opposing facet surfaces, leaving a constant gap of 0.3 mm between each adjacent facet pair. The facet cartilages were linear elastic [35] and meshed using quadratic wedge elements (C3D15). The interaction between FJs was modeled as frictionless tangential behavior and applied a pressure overclosure relationship to define the normal surface contact—pressure at zero gap of 120 MPa [36]. All materials were assumed to be isotropic and linearly elastic. The primary aim was to maintain the simplicity of the model to ensure that the transparency in assessing the effect of each parameter is not lost. The material assumptions were consistent with many existing validated FE spine models [16, 17, 35, 37]. An overview of the material properties that were applied for the full‐spine model development is shown in Table 1. Lumped material properties were considered for the cortical and cancellous bone of the vertebrae, meaning they were combined to be a complete VB, similar for the ground substance and annulus fibrosus of IVDs which were modeled as a combined annulus ring. For each IVD, AF and NP are separated, where AF is linear elastic [37] and NP is treated as a nearly incompressible material (Poisson ratio 0.49) with a cross‐sectional area taking approximately 40% of the entire IVD area [38]. The axial stiffness of ligaments was taken from the literature [15, 39, 40, 41, 42], and the muscle stiffness was calculated using the modulus of elasticity, average cross‐sectional areas, and initial geometrical length from the literature [43, 44, 45, 46].

**TABLE 1:** Summary of material properties used for the full spine FE model.

Spine components	Modulus of elasticity (MPa)/Poisson ratio	References
VB	3000/0.3	Roth et al. [17]
VB posterior	12,000/0.3	Roth et al. [17]
AF	4.2/0.45	Wang et al. [37]
NP	1/0.49	Warren et al. [38]
Facet cartilage	35/0.4	Calvo‐Echenique et al. [35]
Costal cartilage	10.5/0.2	Grünwald et al. [16]
Sternum	12,000/0.3	Same as VB posterior
Rib	11,300/0.3	Grünwald et al. [16]

Ligaments^a^	Axial stiffness (N/mm)	References
ALL _**C, T, L**_	16.00, 81.54, 8.74	Yoganandan et al. [39], Lan et al. [15], Zhang et al. [40]
PLL _**C, T, L**_	25.40, 29.09, 5.83	Yoganandan et al. [39], Lan et al. [15], Zhang et al. [40]
LF _**C, T, L**_	25.00, 68.76, 15.38	Yoganandan et al. [39], Lan et al. [15], Zhang et al. [40]
ISL _**C, T, L**_	7.74, 23.20, 0.19	Mesfar et al. [41], Lan et al. [15], Zhang et al. [40]
SSL _**C, T, L**_	7.74, 31.36, 2.39	Mesfar et al. [41], Lan et al. [15], Zhang et al. [40]
CL _**C, T, L**_	33.60, 1.98, 7.88	Yoganandan et al. [39], Zhang et al. [40]
ITL _**C, T, L**_	1.62, 4.80, 5.43	Yoganandan et al. [39], Lan et al. [15], Zhang et al. [40]
Costotransverse	47.85	Duprey et al. [42]

Musculature	Axial stiffness (N/mm)	References
Serratus posterior^b^_**I, S**_	0.068, 0.068	Borst et al. [43]
Scalene^c^_**A, M, P**_	0.159, 0.124, 0.118	Borst et al. [43]
Iliopsoas	113.44	Hanson et al. [44]
Intercostals	27.926	Kindig et al. [45]
Combined components	9.153	Deeken et al. [46]
Rectus abdominis	0.713	Deeken et al. [46]

^a^ The axial stiffness of ligaments is listed for cervical (C), thoracic (T), and lumbar (L) regions, respectively.

^b^ The axial stiffness of serratus posterior is listed for inferior (I) and superior (S) components.

^c^ The axial stiffness of scalene is listed for anterior, middle, and posterior components.

#### 2.2.2 Elastic Components Attachment

**FIGURE 3:**
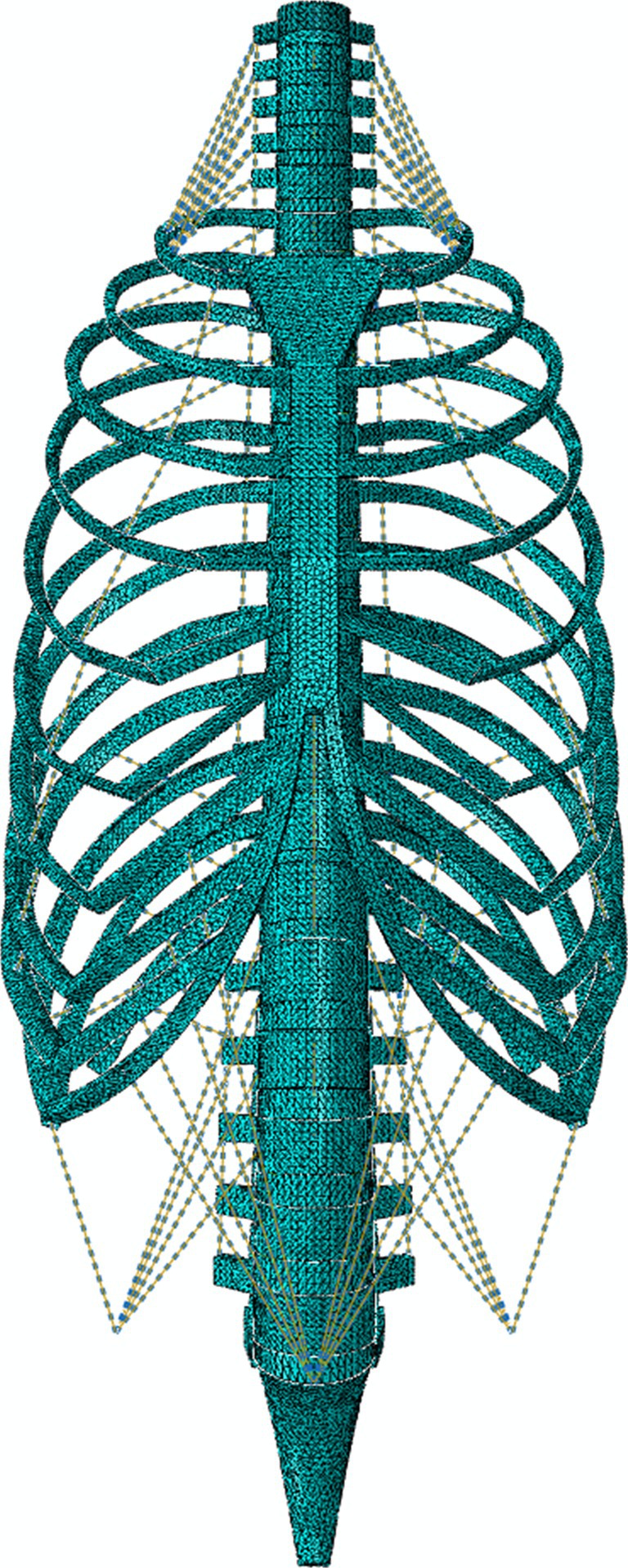
The complete FE spine model with ribcage. The ligaments and musculature composition are presented. Notably, two imaginary pelvis points were positioned for the abdominal muscle attachment.

To aid the joint stability of the spine model, ligaments were modeled as 2‐noded connector elements and attached to the spinal column, including anterior longitudinal (ALL), posterior longitudinal (PLL), ligamentum flavum (LF), interspinous (ISL), supraspinous (SSL), intertransverse (ITL), and capsular ligament (CL). Costovertebral ligaments were defined between the distal end of the rib and the associated thoracic vertebra. Muscles were included for realism and were also modeled using elastic connectors to represent full‐spine muscle effects and attached between the ribcage and the spinal column to limit unrealistic spinal motions. The muscle attachment started at the cervical region involving scalene anterior (C3–C6), middle (C3–C7), and posterior (C5–C7). The muscle at the thoracic region was serratus posterior superior (C7–T3). The muscle configuration was adapted from a validated musculoskeletal model [47] to establish the thoracic and ribcage muscles. Two pelvis point locations were created on both sides of the bottom sacrum (Figure 3) for attaching the abdominal musculatures, including serratus posterior inferior (T11–L2), rectus abdominis (rib 7 to rib 10), and a combined abdominal muscle of obliques (two layers) and transverse abdominis. The muscle attachment ended at the lumbar region, involving the iliopsoas (T12–L3), and the intercostals were also added between the ribs (rib 1 to rib 12).

### 2.3 Tests to Assess the Generic Full‐Spine Model

There is a lack of literature on full‐spine model response [48] and, particularly, with the necessary inclusion and replication of the ribcage stiffening effect [49] and load transmission related to abdominal musculatures [47]. Therefore, in order to assess a full‐spine model, the ideal procedure is to perform tests segment by segment as described by Bellina et al. [18]: material calibration, followed by FE assessments on different spine regions and ribcage. The present study follows this procedure to assess the developed model, thereby evaluating the influence of ligaments, biomechanics of spine regions, and ribcage stiffening effect.

#### 2.3.1 Mesh Sensitivity Analysis

Due to the similarity of three spine regions of the developed generic model, the mesh sensitivity analysis was conducted on the developed lumbar model section to obtain the suitable mesh element size, using five meshing schemes with average element sizes of 1.5, 2, 3, 4, and 5 mm, respectively. In the analysis, the range of motion (ROM) tests were performed by constraining all degrees of freedom of the bottom vertebral surface (L5) and applying 5 Nm pure moment loads on the upper vertebral surface (L1) to simulate backward bending. When the difference between the predicted ROM results by two meshing schemes was less than 5%, the mesh was considered to be converging [4]. To ensure that mesh convergence tests performed based on ROM are adequate, other relevant biomechanical quantities—intradiscal pressure (IDP) and facet joint force (FJF)—were also checked for convergence.

#### 2.3.2 Ligamentous Effect Tests on the Developed Thoracolumbar Model

The mechanical response of the attached ligaments was assessed to test the ligamentous effect on the spinal motion for the selected FSUs of the developed thoracolumbar spine region. A stepwise reduction approach was applied to remove the attached ligaments and FJs in the same sequence as in a previous in vitro study [50, 51], and the test results were compared. ROM tests were conducted on the selected spine region using the boundary conditions as described in the mesh sensitivity analysis with varying loading conditions. The pure moment load was applied to represent four body postures—forward bending (flexion), backward bending (extension), lateral bending, and axial rotation. For the thoracic region, T2‐T3, T6‐T7, and T10‐T11 FSUs were selected for the ROM tests under ±1, ±2.5 Nm pure moment loading conditions as in a previously published in vitro study [50]. For the lumbar region, L4‐L5 FSU was selected for the ROM tests under ±1, ±2.5, ±5, ±7.5, ±10 Nm pure moment loading conditions according to the in vitro study on the same FSU range [51]. Based on the ROM test result comparisons, the ligament materials and the FJ interaction were then calibrated to ensure that the material property assigned provided satisfactory behavior in relevant global and local biomechanical tests.

#### 2.3.3 Biomechanical Assessment of the Full Spine Model

The biomechanical assessments on the full‐spine FE model were conducted segment by segment. The general biomechanical response of the developed lower cervical model was assessed and compared with previous in vitro measurements [52] and a patient‐specific FE model [53]. The ROM tests were conducted on the developed lower cervical model under ±1 Nm pure moment loads. For the thoracic model, since previous in vitro experiments were conducted on human cadaveric specimens [49], this assessment simulated only the passive muscle effect, using an elastic intercostal model where force was exerted only in tension. The ROM tests were conducted for the T1–T12 spinal column model and the complete thoracic model with the ribcage structure to assess the stiffening effect, under ±2 Nm pure moment loads [49]. For the lumbar region, in addition to the ROM tests under ±7.5 Nm pure moment loads, the FJF and IDP were also assessed under the same pure moment loads and compared with previous in vitro [54, 55, 56] and in silico data [10]. Average FJF was calculated throughout the lumbar model FSUs during the ROM tests, using the contact pressures predicted and the facet surface areas. The computation of IDP of the L4–L5 disc was under the 1000 N pure compression to the lumbar model.

#### 2.3.4 Full Spine Muscle Effect Assessment

The attached muscles were modeled using axial stiffness derived from literature [43, 44, 45, 46]. For full‐spine loading experiments, global muscle effects were represented using spring components, allowing forces to be transmitted in both tension and compression to reflect the supportive role of full‐spine muscles in maintaining different body postures. The IDPs were predicted to analyze the global muscle effect on the spinal loading at middle (T6–T7, T7–T8) and lower (T9–T10, T10–T11) spine levels and were compared with the in vivo IDP measurements [57] at the same spine levels. A body weight of 75 kg was assumed, which is similar to the mean body weight of adult samples in the in vivo study [57]. The body mass was distributed along the thoracolumbar spine levels (T1–L5) according to the previous in vitro study [58]. The mass loading positions were defined by anterior offsets from the centers of VBs to represent the centers of gravity of body trunks in the coronal plane at each spine level [34, 58]. The percentage of total body weight (50.8%) was applied to the centers of gravity of body trunk slices as per the previous in silico work [59] to include the weight of the head and upper limbs. The defined centers of gravity of body trunks were connected so that the follower loads [60] are transferred via the axial direction of body trunks. Four postures were simulated [47], including upright standing and lumbar flexion of 30°, with and without 10 kg weight held at each upper limb.

## 3 Results

To ensure consistency in evaluating the comparison between model predictions and referenced data (in vitro and in vivo), the following quantitative criteria were applied throughout the results: excellent match with relative error *r* < 10%; good agreement with *r* between 10% and 15%; satisfactory agreement with *r* between 15% and 20%; poor prediction with *r* > 20%. These thresholds were based on prior validation practices in FE studies [61, 62]. The relative error was calculated as the percentage difference between the model prediction and the reference range.

### 3.1 The Developed Spine Geometry

The geometric dimensions of the generic model were compared with previous studies on the adult spine [1, 23, 29], considering the different age groups of human cadavers employed in the cited studies. The results are provided as supplementary figures (Figures S1–S7, Table S1). In general, the total sagittal vertical height of the developed spine model (501 mm) was 88% of the adult spine (569.4 mm) [29], vertebral body height (VBH) 87.6% on average compared to the age range 55–84 in Busscher et al. [29], 95% on average compared to the age range 19–59 in Panjabi et al. [1, 23], and intervertebral disc height (IVDH) 89% on average compared to the adult IVDH [29]. As during puberty, the standing height is approximately 86% to 95% of the adult height [22], the dimensions of the developed spine model can be considered to represent an adolescent. The developed ribcage model was 96% of the adult ribcage, with rib chord length was 96.7% of the adult rib chord length [25], rib arc length was 98.5% of the adult rib arc length [26], and the difference between the enclosed area of the proposed rib model and that for the adult rib was 6.8% [26].

### 3.2 Mesh Sensitivity Analysis

As shown in Table 2, the percentage differences of the predicted ROMs between five mesh schemes were all within 5% [4]. The results were found to be converging with increasing mesh density. The percentage change between predicted ROMs decreased as the mesh element size reduced, while the execution time increased by a factor of 82. Mesh convergence with average 3 mm edge length elements was further confirmed through FJF and IDP tests, where percentage FJF change (0.34%) and percentage IDP change (0.4%) between mesh schemes was well below the adopted threshold of 5% (see Tables S2 and S3). Therefore, the 3‐mm‐meshing scheme was applied not only for a relatively closer prediction compared with the 1.5‐mm‐meshing scheme, but also for saving the time cost in computation for future FE model assessments and modifications.

**TABLE 2:** Mesh sensitivity analysis based on ROM results using five mesh schemes.

Mesh scheme	1.5 mm	2 mm	3 mm	4 mm	5 mm
Element counts	420,807	187,682	58,904	28,013	15,860
Node counts	636,632	298,314	105,725	50,041	30,613
Predicted ROM (radian)	0.03060	0.03051	0.03042	0.03020	0.02950
Percentage change compared to 1.5 mm mesh scheme	—	0.29%	0.59%	1.31%	3.59%
Percentage change between mesh schemes	0.29%	0.30%	0.73%	2.37%	—

### 3.3 Ligamentous Effect Tests on the Developed Spine Regions

**FIGURE 4:**
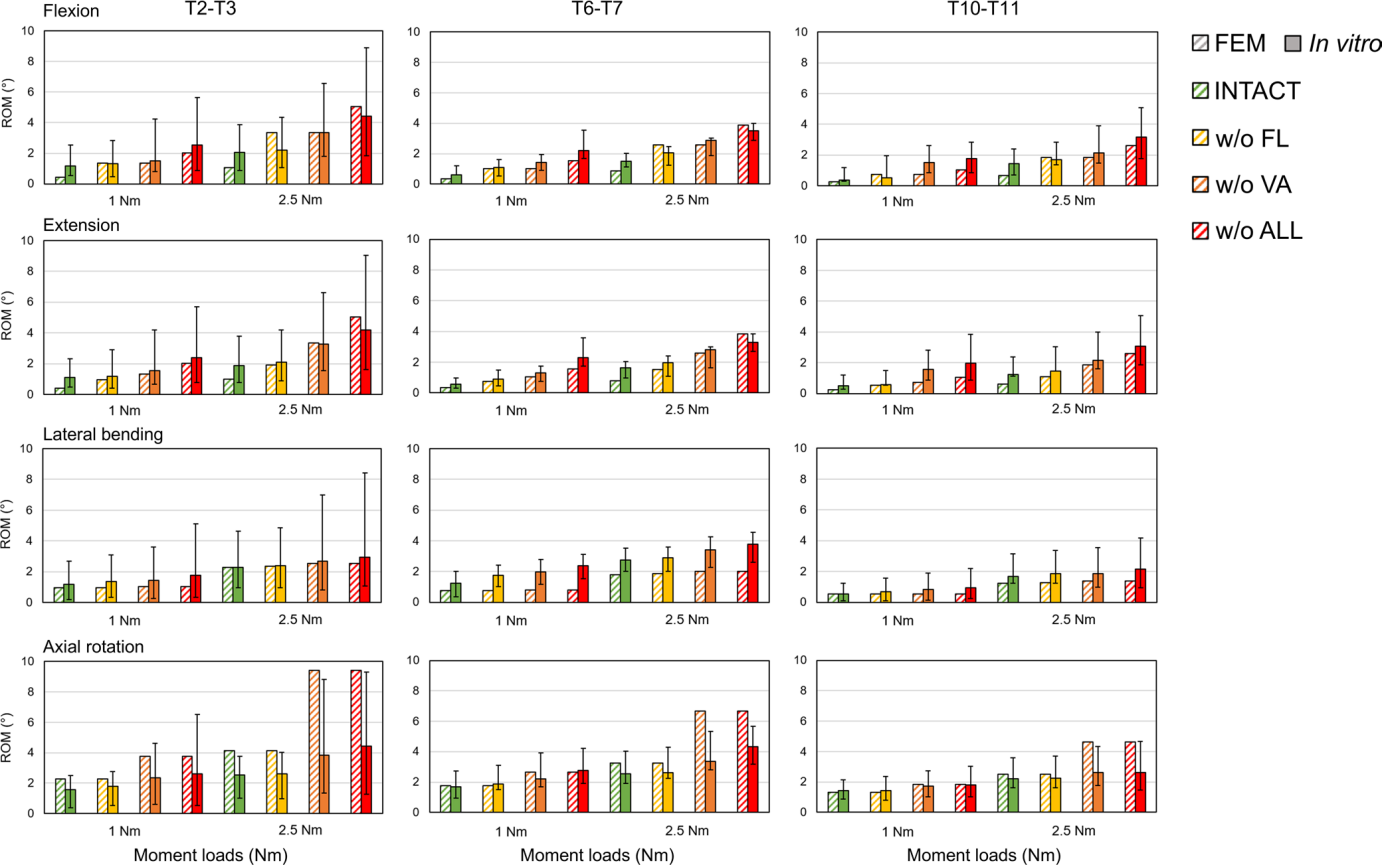
Comparison of the predicted ROMs of thoracic FSUs (T2–T3, T6–T7, T10–T11) in performing four body postures versus in vitro measurements [50] under ±1, ±2.5 Nm pure moment loading.

**FIGURE 5:**
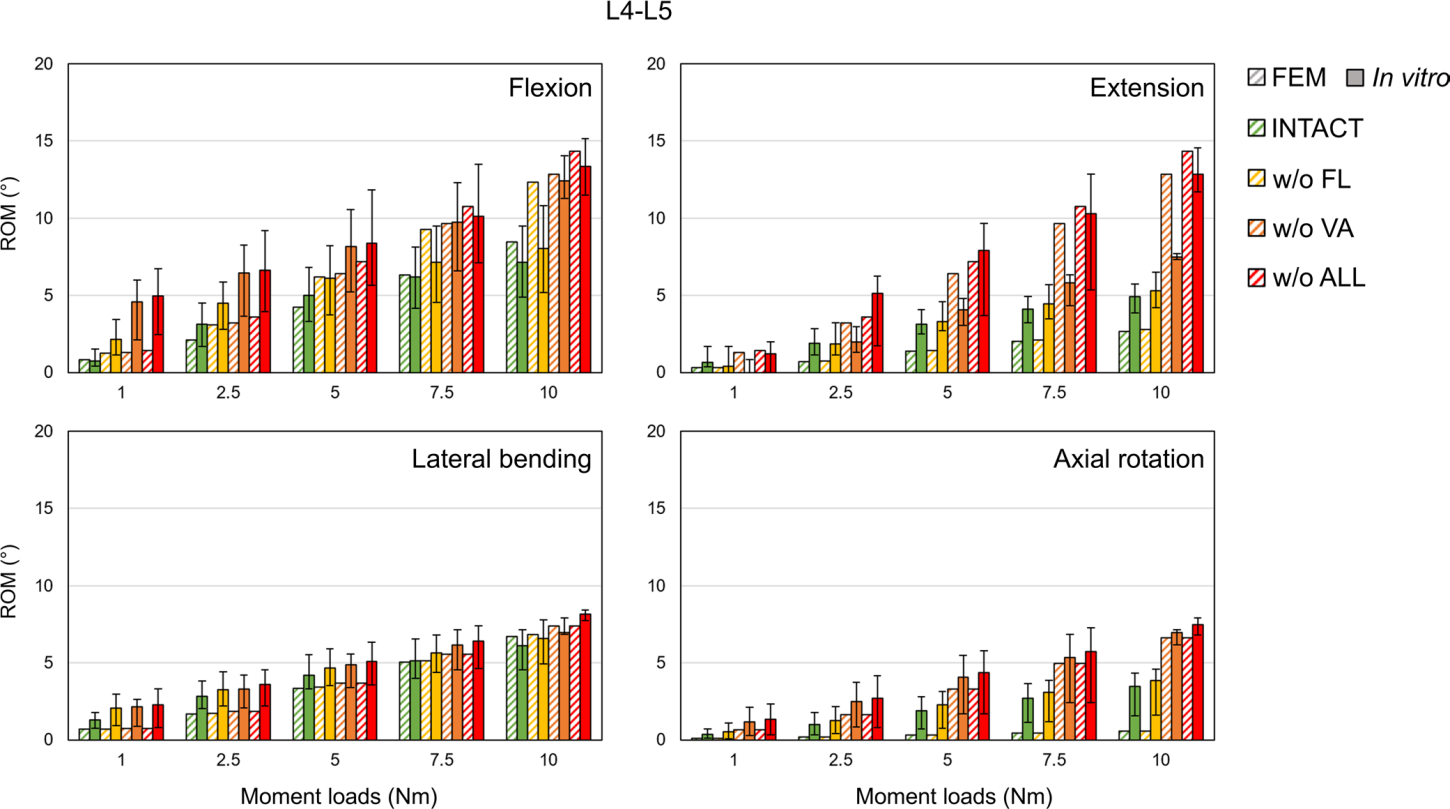
Comparison of the predicted ROMs of lumbar FSU (L4–L5) in performing four body postures versus in vitro measurements [51] under ±1, ±2.5, ±5, ±7.5, ±10 Nm pure moment loading.

**FIGURE 6:**
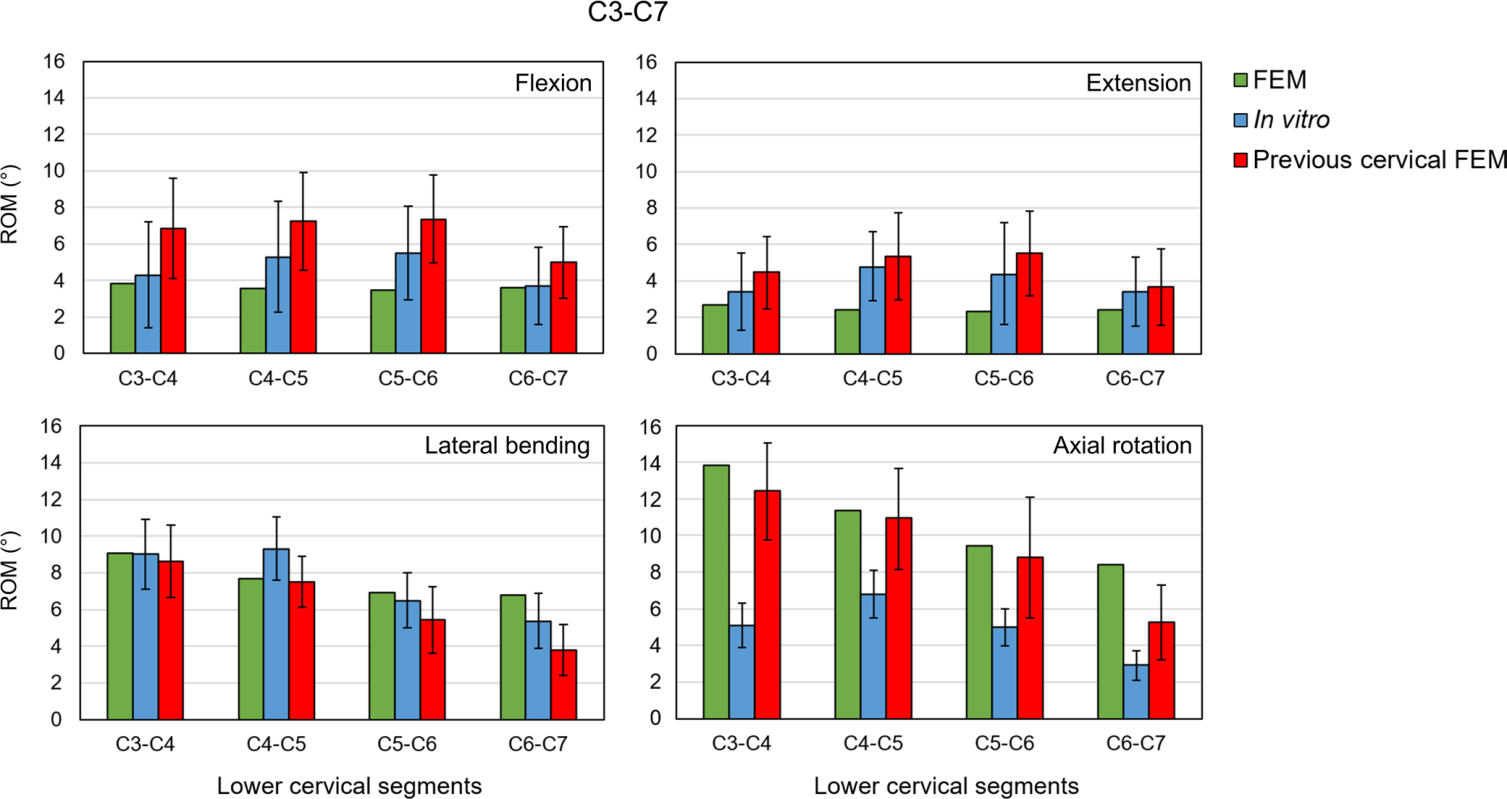
Assessment on the lower cervical region (C3–C7) by predicting global ROMs of four body postures versus in vitro results [52] versus a validated in silico model [53] under ±1 Nm pure moment loading.

**FIGURE 7:**
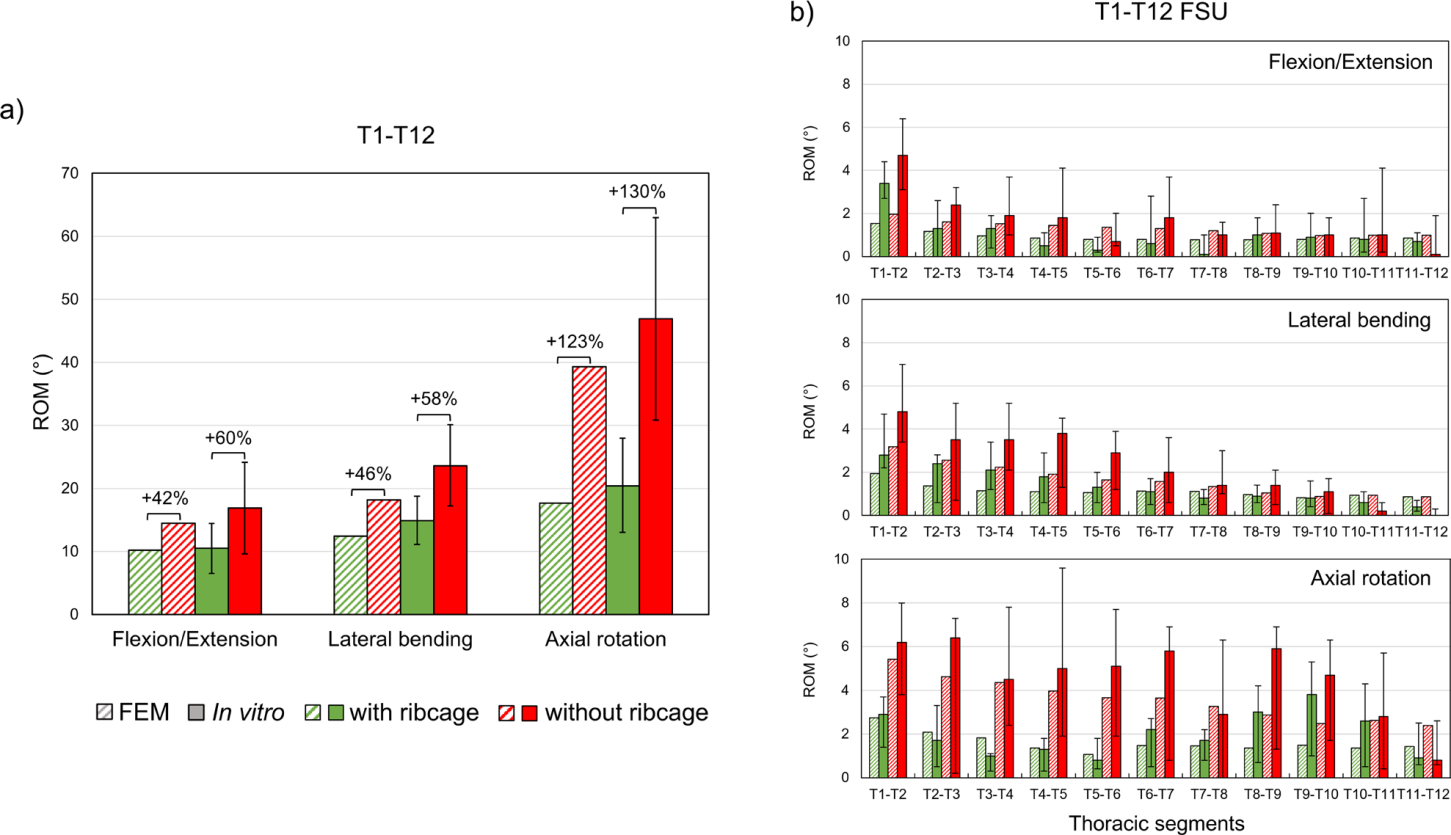
Assessment on the thoracic region (T1–T12) by predicting global (a) and local ROMs (b) of four body postures with/without ribcage versus in vitro measurements [49] under ±2 Nm pure moment loading.

**FIGURE 8:**
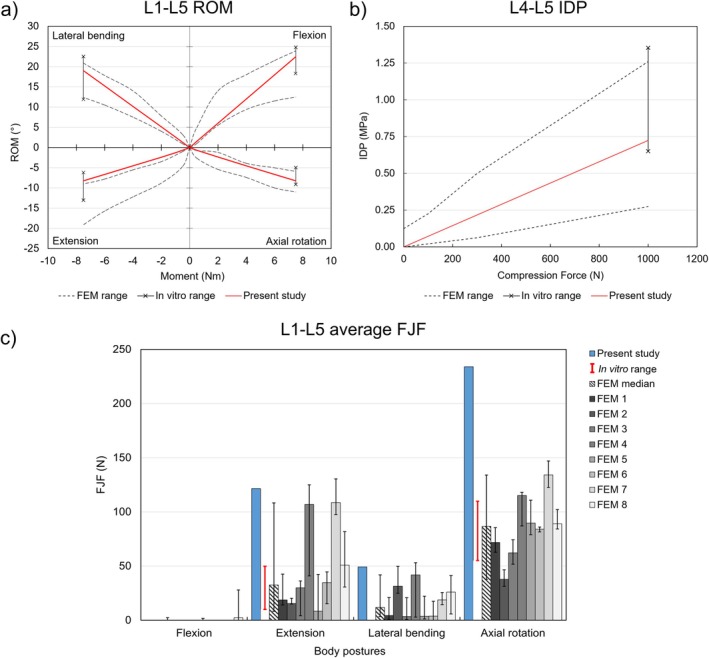
Assessments of the lumbar region (L1–L5). (a) Predicted global ROMs of four body postures under ±7.5 Nm pure moment loading versus in vitro measurements [54]; (b) predicted IDP of L4–L5 intervertebral disc under 1000 N pure compression versus in vitro measurements [55]; (c) predicted average FJFs across lumbar spine levels during ROM tests versus in vitro measurements [56]. The generic lumbar model was also compared with eight FE lumbar models which were presented by Dreischarf [10], showing as the FEM ranges in (a), (b) and eight FEMs in (c).

**FIGURE 9:**
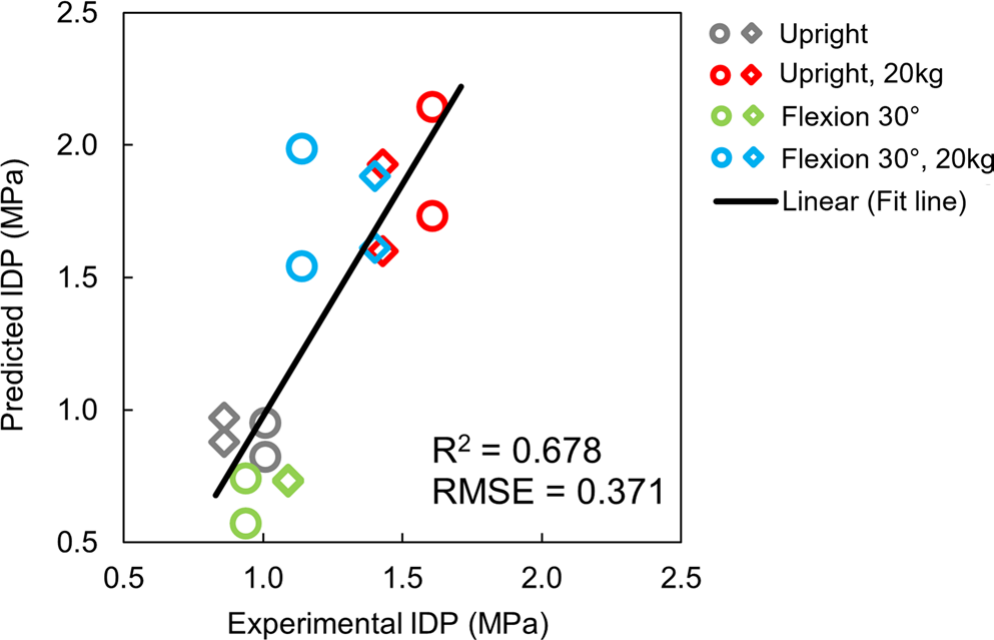
The predicted IDPs by the generic full spine model under static loading tasks. The predictions were plotted against the in vivo measured IDPs [57] for same tested static postures at middle (T6–T7, T7–T8, marked as circles) and lower (T9–T10, T10–T11, marked as diamonds) thoracic levels. Thus, for each in vivo result, there are two predicted IDP values presented.

For the selected spine FSUs, all seven attached ligament types and vertebral arches (VA) were removed step by step for the assessments. Results of four detaching conditions—full intact, without (w/o) FL, w/o VA, and w/o ALL—were presented, which marked the major trends of the predicted ROMs. The comparative design in Figures 4, 5, 6, 7, 8, 9 was inspired by the studies of Bellina et al. [18], Dreischarf et al. [10], and Ignasiak et al. [47], whose work provides a valuable benchmark for spinal modeling performance.

#### 3.3.1 Assessed Thoracic FSUs

Our model generally showed excellent agreement (*r* = 6.57%) with the in vitro reference range [48] (Figure 4). For the four body postures, the predicted ROMs of thoracic FSUs generally increased after the removal of all attached ligaments under 1 Nm and 2.5 Nm moment loads. Under flexion and extension, an excellent match (*r* = 8.38%) to the in vitro measurements was presented, with similar trends observed in ROM variation. Minor variations were observed in the predicted ROMs during lateral bending tests, with tests on upper (T2–T3) and lower (T10–T11) FSUs all within in vitro ranges, and tests on the middle FSU (T6‐T7) showing relatively poor predictions (*r* = 19.64%). The most significant ROM variations were observed during axial rotations (51.1% increase in ROM) after removing VA, which was caused by removing the facet joint interactions.

#### 3.3.2 Assessed Lumbar FSU

Our model generally showed a satisfactory agreement (*r* = 16.44%) with the in vitro measurements [51] (Figure 5), except for the poor predictions during extension (*r* = 26.7%), especially after removing VA and increasing the moment load to 5 Nm. At higher moment loading, the removal of VAs and related facet joints led to vertebral bending primarily depending on the stability of IVDs, resulting in significantly high ROMs predicted for extension, which were similar to the ROMs predicted during flexion. For four body postures, the spinal motion of lumbar FSU generally improved after removing all the attached ligaments under moment loads from 1 to 10 Nm. Except for flexion, the removal of FL barely affected the spinal motion of lumbar FSU, while the removal of VA significantly increased the predicted ROMs. Before the removal of VA, the employed facet joint contact definition led to limited axial rotations for all loading tasks, which were generally 49.92% below the in vitro corridors.

### 3.4 Biomechanical Assessments of the Full Spine

#### 3.4.1 Cervical FE Model Assessment

The local ROM predictions of the lower cervical FSUs (Figure 6) showed an excellent agreement (*r* = 1.59%) with the in vitro measurements [52] except for the axial rotation cases. Comparison with the previously validated patient‐specific FE model [53] indicated that the present cervical model is relatively stiffer in the flexion and extension. For the axial rotation, the previous in silico model and the present study shared the similar trend in ROM prediction, which was perfectly matched (*r* = 9.74%) but both exceeded the mean ROM measured by the in vitro study.

#### 3.4.2 Thoracic FE Model Assessment

With and without ribcage, the global ROM predictions of the thoracic model showed an excellent agreement (*r* = 5.74%) with the in vitro measurements [49]. According to the percentage loss of ROMs noted on bars (Figure 7a), the present study showed a similar ribcage stiffening effect as the in vitro results. Inclusion of ribcage had a strong effect on restricting the spinal motion in all three directions, especially in the axial rotation (123%). For the monosegmental ROM, the in vitro and present study showed similar descending trends in ROM variations (Figure 7b). The highest flexibility was detected at T1–T2 FSU, while the lowest flexibility was detected at the lower thoracic segments (T9–T10 to T11–T12). However, the flexibility of the present model was more evenly distributed throughout the thoracic FSUs (T1–T12) compared to the in vitro local ROMs with and without the ribcage.

#### 3.4.3 Lumbar FE Model Assessment

The simulation results were compared to previous in vitro studies and eight previous FE models [10]. The ROM predictions were all within the in vitro measurements (vertical intervals, Figure 8a) [54] and perfectly matched the in silico corridors (*r* = 3.15%) (dashed curves, Figure 8a) in flexion, lateral bending, and axial rotation, except for the extension, which was slightly underestimated (*r* = 8.56%). The predicted IDP (0.72 MPa) showed a good agreement (*r* = 11.32%) with both in vitro data [55] and was within the in silico corridor (Figure 8b). The predicted average FJF across lumbar spine levels showed a satisfactory agreement (*r* = 17.62%) with several in silico models in flexion (average 0 N), extension, and lateral bending, but the average FJF during axial rotation was significantly overestimated (*r* = 154%). Overall, our predicted FJFs followed a similar force transmission pattern as previous in silico models, but all exceeded the in vitro FJF range [56] (Figure 8c). The average FJF was minimal during flexion, with the highest FJF occurring during axial rotation.

### 3.5 Full Spine Muscle Effect Assessment

The predicted IDPs showed a satisfactory correlation (*R* ^2^ = 0.678) with the experimental IDP values measured in vivo [57]. Generally, IDPs of the middle thoracic levels were relieved in spine flexions compared to the upright standing postures, while IDPs of the lower thoracic levels were close between the upright standing and flexion postures, especially under 20 kg of extra load. The average difference between values predicted by the generated spine model and the experimental in vivo measurements was estimated in root mean square error (RMSE), which was 0.371, showing that the generic model was satisfactorily predicting the spinal loading.

## 4 Discussion

### 4.1 Generic Spine Model Development

This study demonstrated that a FE model based on average parametric data can satisfactorily replicate the structural behavior of the spine and represent realistic biomechanical responses in various loading simulations. Additionally, the inclusion of musculature and ribcage allows the model to replicate in vivo‐like biomechanics, such as the stiffening effect and muscle‐induced load transmission, which are crucial for analyzing surgical simulations. This full‐spine model with ribcage was developed using data from previous studies on spine morphology [20, 21, 24, 27, 28, 29, 33] and spinal joints [35, 36], ligaments [15, 39, 40], and muscles [43, 44, 45, 46]. Due to the lack of literature on normal adolescent full‐spine modeling, our generic model used average adult spine morphological parameters to derive and simplify the adolescent spine geometry, including the ribcage and associated muscles with lower complexity compared to patient‐specific models, while still capturing the biomechanical behaviors of interest.

The developed full‐spine model showed several advantages over current spine modeling techniques, which have recently gained popularity in clinical biomechanics studies and applications [63]. Compared to musculoskeletal models that use multiple rigid bodies to represent vertebrae connected by mechanical joints [47, 64], our model contained deformable materials within FE, allowing examination of deformation, strain, stress, and other mechanical parameters for spinal components, which can be easily monitored. Compared to previous FE models, this study conducted a full‐spine ROM analysis, which was not included in Jia et al. [65]; investigated the role of ligaments and spinal joints via a biofidelic vertebral posterior design, which was absent in Grünwald et al. [16]; assessed the impact of including ribcage in a full‐spine model, which was not considered in Roth et al. [17]; and finally, evaluated the developed muscle configuration, which is typically absent in most existing full‐spine FE models [16, 17, 18, 65].

When considering differences between adult and adolescent spine models, one valid concern is whether morphological relationships established in adult spines can be directly applied to adolescent cases, given the distinct stages of body growth [21, 22]. The present study does not entirely eliminate this concern; our parametric modelling approach did not incorporate regression analyses on multiple morphology‐dependent parameters specific to adolescents. Our design process was simplified CAD design based on derived spine morphology. However, since the geometric dimensions of the developed adolescent spine were demonstrated to be approaching skeletal maturity, the absence of regression analysis based parameters only led to a limited degree of mismatch between spinal components.

### 4.2 Generic Full Spine Model Assessment

#### 4.2.1 Mesh Sensitivity Analysis

Some in silico patient‐specific spine models have employed finer meshes of element size 0.5 mm or 1 mm [40]; our parametric spine model showed that a coarser mesh effectively ensured convergence, but this is related to our simplified parametric design of the model. Patient‐specific models require finer meshes to avoid the accumulation of shape and volume errors and to prevent stress concentrations caused by small anatomical structures.

#### 4.2.2 Ligamentous Effect Tests on the Developed Thoracolumbar Model

The tests conducted on the selected thoracic and lumbar FSUs effectively demonstrated the role of the attached elastic components and spinal joints in maintaining spine stability and flexibility. For four simulated body postures on all tested FSUs, stepwise removal of ligaments and vertebral arches resulted in expected increments in ROM, with most outcomes consistent with previous in vitro studies [50, 51]. Notably, the facet joint model of our generic spine had a significant influence on the axial rotation in both thoracic and lumbar regions, causing the largest ROM variations among all numerical experiments. This was especially evident when comparing ROMs before and after removing the vertebral arches. For the thoracic region, the predicted ROMs exceeded the in vitro range once the vertebral arches were removed, while for the lumbar region, the spinal motion was heavily restricted by facet contact elements.

It was also noticeable that after the removal of facet joints in our models, spinal motion depended primarily on the stiffness of IVDs, resulting in a similar range of forward and backward bending motions for all thoracic and lumbar FSUs. For the lumbar spine, our result did not match the in vitro measurements as extension typically showed a lower ROM after the removal of facet joints compared to flexion under the same loading conditions [51]. This discrepancy can be attributed to the properties assigned to the annulus fibrosus [66], as well as the comparable height differences between anterior and posterior vertebral bodies.

#### 4.2.3 Full Spine Biomechanical Assessments

The model validation across different spine regions showed promising results compared to in vitro measurements. The model development approach employed resulted in the lower cervical spine model being less geometrically precise with respect to vertebral bodies and facet joints. Its inclusion was intended to establish the upper thoracic muscles and compensate for the thoracolumbar spine motion for future studies. Since most previous studies delineated load–displacement relationships of the cervical spine, including the upper C1 and C2 vertebrae [67, 68], the overall stiffness of our lower cervical model (C3–C7) was adjusted by varying material properties to achieve a satisfactory ROM that aligns with in vitro data. This indicates that the lower cervical model is sufficient for including neck muscles and representing the cervical spine motions.

For the thoracic region, the generic model showed excellent biomechanical performance in predicting both global and local ROMs compared to in vitro measurements [49]. The inclusion of the ribcage significantly restricted spinal motions, particularly in axial rotation, replicating the st iffening effect in vivo. Jia et al. [65] attempted to study the influence of the ribcage on the static and dynamic stability responses of both normal and scoliotic spines but did not consider the influence of neck and abdominal muscles for conducting ROM tests.

Model assessment on the lumbar region showed that the geometric design and joint connections of the developed generic spine model would result in similar mechanical responses compared to the previous in vitro [54, 55, 56] and in silico [10] studies, with the exception of significant overestimation in axial rotation FJFs. The overestimation in FJFs was also observed in previous in silico models [10] with considerable variations between eight tested models in all loading tasks, which suggests that FJF predictions are highly sensitive to the facet joint contact definition—initial joint gap, cartilage thickness, and contact area. A previous FE study [69] demonstrated that smaller initial facet gaps with constant cartilage thickness generally led to greater total contact areas and higher predicted contact forces. In this study, the facet joint was defined by two parallel surfaces with a constant thickness of 0.2 mm cartilage layers in total and a 0.3 mm joint gap. This definition resulted in a larger effective contact areas (533.96 mm^2^) compared to the realistic facet contacts (300–400 mm^2^) [69], a thinner cartilage thickness compared to the mean lumbar facet cartilage thickness (0.57 mm) [69], and a smaller gap compared to the previously reported facet models (0.5 mm) [36]. As hypothesized by Dreischarf et al. [10], the median of pooled predictions from multiple numerical models was more consistent with in vitro measurements than individual results, emphasizing the need for caution when interpreting individual FJF predictions. These should be viewed primarily as a rough understanding of the internal load transmission throughout the lumbar model rather than precise measures.

#### 4.2.4 Full Spine Muscle Effect Assessment

The muscle configuration was assessed for the generic full‐spine model, which correlated well with in vivo measurements [57], particularly for the lower thoracic levels during static loading tasks (*R* ^2^ = 0.77). The predicted IDPs at middle thoracic levels also showed a good correlation to the experimental values (*R* ^2^ = 0.7), except for the ones with 20 kg extra loadings. This discrepancy could be attributed to the absence of muscle activation under high‐load conditions in the model. Nevertheless, the result suggested that the global muscle effect was effectively represented by the spring elements attached to the spine. However, musculoskeletal geometry and function differ between adolescents and adults due to ongoing growth and maturation [20, 30]. Since detailed in vivo datasets on muscle morphology or force pathways in adolescents remain scarce in the literature, adult reference data were used as a preliminary benchmark to qualitatively evaluate the plausibility of the muscle configuration in this study, rather than as direct validation for adolescent‐specific values. The comparison is an exploratory step in understanding the muscle‐stabilized spinal behavior. Results for this assessment, as well as previous assessments on the full‐spine biomechanics, only represent the normal healthy adolescent spine developed in this study rather than other spine models within the physiological or pathological conditions. For clinical cases, further model adaptations would be required to account for specific anatomical and biomechanical variations. This includes modifying vertebral morphology, IVD properties, and muscular contribution strategies that reflect the pathological condition under investigation.

Also, this assessment is insufficient to comprehensively understand the overall muscle effect on the full‐spine model. In reality, muscle mechanics are more complex, as muscles cannot only be elastically stretched but also actively contract and generate force that compromises the internal and external responses of the spine [70]. In vivo‐like spinal FE models [71, 72] are better suited for investigating realistic muscle actions, as these models involve a more comprehensive anatomical structure of the human body (head, limbs, internal cavities) to evaluate the global dynamic characteristics of the spine in the time domain. Simulating muscles as purely spring components ignores the active contraction and time‐dependent mechanical behavior of muscles [73], and the critical role of muscles in dynamic motion biomechanics is not investigated in this study.

### 4.3 Clinical Relevance

The developed generic full‐spine model will be employed to create abnormal spine geometries to test treatment options. Of particular interest to the authors is the creation of different scoliotic patterns encountered in clinical practice. Thus, future study will investigate a wide range of scoliosis cases subjected to varying surgical techniques. The generated scoliotic models will be employed to compare different surgical correction techniques on the same scoliotic case and to compare the performance of one surgical conduct across different scoliotic cases. This will enable the development of an instructive reference for spinal surgeons to assess the merits and feasibility of different treatment approaches.

## Ethics Statement

The authors have nothing to report.

## Conflicts of Interest

The authors declare no conflicts of interest.

## Supporting information


**Data S1:** Supporting Information.

## Data Availability

The data used to generate the model was obtained from studies cited in the study. The data that support the findings of this study is available from the corresponding author upon reasonable request.

## References

[1] M. M. Panjabi , V. Goel , T. Oxland , et al., “Human Lumbar Vertebrae: Quantitative Three‐Dimensional Anatomy,” Spine 17, no. 3 (1992): 299–306, 10.1097/00007632-199203000-00010. 1566168

[2] M. M. Panjabi and A. A. White, III , “Basic Biomechanics of the Spine,” Neurosurgery 7, no. 1 (1980): 76–93, 10.1227/00006123-198007000-00014. 7413053

[3] M. M. Panjabi , T. Miura , P. A. Cripton , J. L. Wang , A. S. Nain , and C. DuBois , “Development of a System for In Vitro Neck Muscle Force Replication in Whole Cervical Spine Experiments,” Spine 26, no. 20 (2001): 2214–2219, 10.1097/00007632-200110150-00012. 11598511

[4] A. C. Jones and R. K. Wilcox , “Finite Element Analysis of the Spine: Towards a Framework of Verification, Validation and Sensitivity Analysis,” Medical Engineering & Physics 30, no. 10 (2008): 1287–1304, 10.1016/j.medengphy.2008.09.006. 18986824

[5] P. R. Loughenbury , A. I. Tsirikos , and N. W. Gummerson , “Spinal Biomechanics–Biomechanical Considerations of Spinal Stability in the Context of Spinal Injury,” Orthopaedics and Traumatology 30, no. 5 (2016): 369–377, 10.1016/j.mporth.2016.07.010.

[6] M. Fagan , S. Julian , and A. Mohsen , “Finite Element Analysis in Spine Research,” Proceedings of the Institution of Mechanical Engineers, Part H: Journal of Engineering in Medicine 216, no. 5 (2002): 281–298, 10.1243/09544110260216568. 12365787

[7] S. L. Gould , L. Cristofolini , G. Davico , and M. Viceconti , “Computational Modelling of the Scoliotic Spine: A Literature Review,” International Journal for Numerical Methods in Biomedical Engineering 37, no. 10 (2021): e3503, 10.1002/cnm.3503. 34114367 PMC8518780

[8] K. Gunasekaran , K. S. Basaruddin , N. A. Muhayudin , and A. R. Sulaiman , “Corrective Mechanism Aftermath Surgical Treatment of Spine Deformity due to Scoliosis: A Systematic Review of Finite Element Studies,” BioMed Research International 2022, no. 1 (2022): 5147221, 10.1155/2022/5147221. 35898687 PMC9314159

[9] J. P. Little , M. T. Izatt , R. D. Labrom , G. N. Askin , and C. J. Adam , “An FE Investigation Simulating Intra‐Operative Corrective Forces Applied to Correct Scoliosis Deformity,” Scoliosis 8 (2013): 1–14, 10.1186/1748-7161-8-9. 23680391 PMC3680303

[10] M. Dreischarf , T. Zander , A. Shirazi‐Adl , et al., “Comparison of Eight Published Static Finite Element Models of the Intact Lumbar Spine: Predictive Power of Models Improves When Combined Together,” Journal of Biomechanics 47, no. 8 (2014): 1757–1766, 10.1016/j.jbiomech.2014.04.002. 24767702

[11] J. Zheng , Y. Yang , S. Lou , D. Zhang , and S. Liao , “Construction and Validation of a Three‐Dimensional Finite Element Model of Degenerative Scoliosis,” Journal of Orthopaedic Surgery and Research 10 (2015): 1–7, 10.1186/s13018-015-0334-1. 26704779 PMC4690237

[12] T. Lodygowski , M. Wierszycki , and M. Ogurkowska , “Three‐Dimensional Nonlinear Finite Element Model of Lumbar Intervertebral Disc,” Acta of Bioengineering and Biomechanics 7, no. 2 (2005): 29.

[13] K. Szkoda‐Poliszuk , M. Żak , and C. Pezowicz , “Finite Element Analysis of the Influence of Three‐Joint Spinal Complex on the Change of the Intervertebral Disc Bulge and Height,” International Journal for Numerical Methods in Biomedical Engineering 34, no. 9 (2018): e3107, 10.1002/cnm.3107. 29799170

[14] M. A. Liebschner , D. L. Kopperdahl , W. S. Rosenberg , and T. M. Keaveny , “Finite Element Modeling of the Human Thoracolumbar Spine,” Spine 28, no. 6 (2003): 559–565, 10.1097/01.BRS.0000049923.27694.47. 12642762

[15] C.‐C. Lan , C.‐S. Kuo , C.‐H. Chen , and H.‐T. Hu , “Finite Element Analysis of Biomechanical Behavior of Whole Thoraco‐Lumbar Spine With Ligamentous Effect,” Changhua Journal of Medicine 11, no. 1 (2013): 26–41.

[16] A. T. Grünwald , S. Roy , A. Alves‐Pinto , and R. Lampe , “Assessment of Adolescent Idiopathic Scoliosis From Body Scanner Image by Finite Element Simulations,” PLoS One 16, no. 2 (2021): e0243736, 10.1371/journal.pone.0243736. 33566808 PMC7875351

[17] A. K. Roth , A. S. Beheshtiha , R. van der Meer , et al., “Validation of a Finite Element Model of the Thoracolumbar Spine to Study Instrumentation Level Variations in Early Onset Scoliosis Correction,” Journal of the Mechanical Behavior of Biomedical Materials 117 (2021): 104360, 10.1016/j.jmbbm.2021.104360. 33588212

[18] E. Bellina , M. E. Laurino , A. Perego , et al., “Assessment of a Fully‐Parametric Thoraco‐Lumbar Spine Model Generator With Articulated Ribcage,” Journal of Biomechanics 164 (2024): 111951, 10.1016/j.jbiomech.2024.111951. 38310005

[19] M. Kurutz and L. Oroszváry , “Finite Element Modeling and Simulation of Healthy and Degenerated Human Lumbar Spine,” in Finite Element Analysis—From Biomedical Applications to Industrial Developments (InTech, 2012), 193–216, 10.5772/37384.

[20] A. Frostell , R. Hakim , E. P. Thelin , P. Mattsson , and M. Svensson , “A Review of the Segmental Diameter of the Healthy Human Spinal Cord,” Frontiers in Neurology 7 (2016): 238, 10.3389/fneur.2016.00238. 28066322 PMC5179522

[21] F. Canavese and A. Dimeglio , “Normal and Abnormal Spine and Thoracic Cage Development,” World Journal of Orthopedics 4, no. 4 (2013): 167–174, 10.5312/wjo.v4.i4.167. 24147251 PMC3801235

[22] A. Dimeglio , F. Canavese , and F. Bonnel , “Normal Growth of the Spine and Thorax,” in The Growing Spine: Management of Spinal Disorders in Young Children (Springer Berlin Heidelberg, 2016), 47–82, 10.1007/978-3-662-48284-1_4.

[23] M. M. Panjabi , K. Takata , V. Goel , et al., “Thoracic Human Vertebrae Quantitative Three‐Dimensional Anatomy,” Spine 16, no. 8 (1991): 888–901, 10.1097/00007632-199108000-00006. 1948374

[24] M. M. Panjabi , T. Oxland , K. Takata , V. Goel , J. Duranceau , and M. Krag , “Articular Facets of the Human Spine Quantitative Three‐Dimensional Anatomy,” Spine 18, no. 10 (1993): 1298–1310, 10.1097/00007632-199308000-00009. 8211362

[25] S. A. Holcombe , S. C. Wang , and J. B. Grotberg , “Modeling Female and Male Rib Geometry With Logarithmic Spirals,” Journal of Biomechanics 49, no. 13 (2016): 2995–3003, 10.1016/j.jbiomech.2016.07.021. 27497501

[26] J. Dansereau and I. A. Stokes , “Measurements of the Three‐Dimensional Shape of the Rib Cage,” Journal of Biomechanics 21, no. 11 (1988): 893–901, 10.1016/0021-9290(88)90127-3. 3075610

[27] J. Burgos , C. Barrios , G. Mariscal , A. Lorente , and R. Lorente , “Non‐Uniform Segmental Range of Motion of the Thoracic Spine During Maximal Inspiration and Exhalation in Healthy Subjects,” Frontiers in Medicine 8 (2021): 699357, 10.3389/fmed.2021.699357. 34527680 PMC8435595

[28] K. T. Johnson , W. N. al‐Holou , R. C. E. Anderson , et al., “Morphometric Analysis of the Developing Pediatric Cervical Spine,” Journal of Neurosurgery: Pediatrics 18, no. 3 (2016): 377–389, 10.3171/2016.3.PEDS1612. 27231821

[29] I. Busscher , J. J. W. Ploegmakers , G. J. Verkerke , and A. G. Veldhuizen , “Comparative Anatomical Dimensions of the Complete Human and Porcine Spine,” European Spine Journal 19 (2010): 1104–1114, 10.1007/s00586-010-1326-9. 20186441 PMC2900026

[30] F. Canavese , A. Dimeglio , F. Bonnel , et al., “Thoracic Cage Volume and Dimension Assessment by Optoelectronic Molding in Normal Children and Adolescents During Growth,” Surgical and Radiologic Anatomy 41 (2019): 287–296, 10.1007/s00276-018-2164-4. 30560403

[31] J. Razzouk , M. Kricfalusi , T. Case , et al., “Surface Anatomical Landmarks for Spine Surgery: A CT‐Based Study of the Sternal Notch and Sternal Angle in 1,035 Patients,” Journal of Clinical Neuroscience 118 (2023): 46–51, 10.1016/j.jocn.2023.08.024. 37866208

[32] E. Been , S. Shefi , and M. Soudack , “Cervical Lordosis: The Effect of Age and Gender,” Spine Journal 17, no. 6 (2017): 880–888, 10.1016/j.spinee.2017.02.007. 28254673

[33] C. A. Giglio and J. B. Volpon , “Development and Evaluation of Thoracic Kyphosis and Lumbar Lordosis During Growth,” Journal of Children's Orthopaedics 1, no. 3 (2007): 187–193, 10.1007/s11832-007-0033-5. PMC2656721 19308494

[34] J. M. Warren , L. A. Hey , and A. P. Mazzoleni , “A Finite Element Study of the Relationship Between Upper Body Weight and the Loads Experienced by the Human Lumbosacral Spine, and Fusion Instrumentation, in a Standing Upright Posture,” Biomedical Engineering Advances 2 (2021): 100023, 10.1016/j.bea.2021.100023.

[35] A. Calvo‐Echenique , J. Cegoñino , R. Chueca , and A. Pérez‐del Palomar , “Stand‐Alone Lumbar Cage Subsidence: A Biomechanical Sensitivity Study of Cage Design and Placement,” Computer Methods and Programs in Biomedicine 162 (2018): 211–219, 10.1016/j.cmpb.2018.05.022. 29903488

[36] M. Mengoni , “Biomechanical Modelling of the Facet Joints: A Review of Methods and Validation Processes in Finite Element Analysis,” Biomechanics and Modeling in Mechanobiology 20, no. 2 (2021): 389–401, 10.1007/s10237-020-01403-7. 33221991 PMC7979651

[37] L. Wang , B. Zhang , S. Chen , X. Lu , Z. Y. Li , and Q. Guo , “A Validated Finite Element Analysis of Facet Joint Stress in Degenerative Lumbar Scoliosis,” World Neurosurgery 95 (2016): 126–133, 10.1016/j.wneu.2016.07.106. 27521732

[38] J. M. Warren , A. P. Mazzoleni , and L. A. Hey , “Development and Validation of a Computationally Efficient Finite Element Model of the Human Lumbar Spine: Application to Disc Degeneration,” International Journal of Spine Surgery 14, no. 4 (2020): 502–510, 10.14444/7066. 32986570 PMC7478021

[39] N. Yoganandan , S. Kumaresan , and F. A. Pintar , “Geometric and Mechanical Properties of Human Cervical Spine Ligaments,” Journal of Biomechanical Engineering 122, no. 6 (2000): 623–629, 10.1115/1.1322034. 11192384

[40] Q. Zhang , Y. Zhang , T. E. Chon , J. S. Baker , and Y. Gu , “Analysis of Stress and Stabilization in Adolescent With Osteoporotic Idiopathic Scoliosis: Finite Element Method,” Computer Methods in Biomechanics and Biomedical Engineering 26, no. 1 (2023): 12–24, 10.1080/10255842.2022.2044803. 35393912

[41] W. Mesfar and K. Moglo , “Effect of the Transverse Ligament Rupture on the Biomechanics of the Cervical Spine Under a Compressive Loading,” Clinical Biomechanics 28, no. 8 (2013): 846–852, 10.1016/j.clinbiomech.2013.07.016. 23972374

[42] S. Duprey , D. Subit , H. Guillemot , and R. W. Kent , “Biomechanical Properties of the Costovertebral Joint,” Medical Engineering & Physics 32, no. 2 (2010): 222–227, 10.1016/j.medengphy.2009.12.001. 20036178

[43] J. Borst , P. A. Forbes , R. Happee , and D. J. ( H. E. J.) Veeger , “Muscle Parameters for Musculoskeletal Modelling of the Human Neck,” Clinical Biomechanics 26, no. 4 (2011): 343–351, 10.1016/j.clinbiomech.2010.11.019. 21247677

[44] P. Hanson , P. Aagaard , and S. P. Magnusson , “Biomechanical Properties of Isolated Fascicles of the Iliopsoas and Achilles Tendons in African American and Caucasian Men,” Annals of Anatomy‐Anatomischer Anzeiger 194, no. 5 (2012): 457–460, 10.1016/j.aanat.2012.03.007. 22583513

[45] M. Kindig , Z. Li , R. Kent , and D. Subit , “Effect of Intercostal Muscle and Costovertebral Joint Material Properties on Human Ribcage Stiffness and Kinematics,” Computer Methods in Biomechanics and Biomedical Engineering 18, no. 5 (2015): 556–570, 10.1080/10255842.2013.820718. 23947597

[46] C. R. Deeken and S. P. Lake , “Mechanical Properties of the Abdominal Wall and Biomaterials Utilized for Hernia Repair,” Journal of the Mechanical Behavior of Biomedical Materials 74 (2017): 411–427, 10.1016/j.jmbbm.2017.05.008. 28692907

[47] D. Ignasiak , S. Dendorfer , and S. J. Ferguson , “Thoracolumbar Spine Model With Articulated Ribcage for the Prediction of Dynamic Spinal Loading,” Journal of Biomechanics 49, no. 6 (2016): 959–966, 10.1016/j.jbiomech.2015.10.010. 26684431

[48] I. El Bojairami , K. El‐Monajjed , and M. Driscoll , “Development and Validation of a Timely and Representative Finite Element Human Spine Model for Biomechanical Simulations,” Scientific Reports 10, no. 1 (2020): 21519, 10.1038/s41598-020-77469-1. 33298988 PMC7725813

[49] C. Liebsch , N. Graf , K. Appelt , and H. J. Wilke , “The Rib Cage Stabilizes the Human Thoracic Spine: An In Vitro Study Using Stepwise Reduction of Rib Cage Structures,” PLoS One 12, no. 6 (2017): e0178733, 10.1371/journal.pone.0178733. 28570671 PMC5453693

[50] H.‐J. Wilke , S. Grundler , C. Ottardi , C. E. Mathew , B. Schlager , and C. Liebsch , “In Vitro Analysis of Thoracic Spinal Motion Segment Flexibility During Stepwise Reduction of All Functional Structures,” European Spine Journal 29 (2020): 179–185, 10.1007/s00586-019-06196-7. 31664565

[51] F. Heuer , H. Schmidt , and H.‐J. Wilke , “Stepwise Reduction of Functional Spinal Structures Increase Disc Bulge and Surface Strains,” Journal of Biomechanics 41, no. 9 (2008): 1953–1960, 10.1016/j.jbiomech.2006.03.016. 18501361

[52] M. M. Panjabi , J. J. Crisco , A. Vasavada , et al., “Mechanical Properties of the Human Cervical Spine as Shown by Three‐Dimensional Load–Displacement Curves,” Spine 26, no. 24 (2001): 2692–2700, 10.1097/00007632-200112150-00012. 11740357

[53] M. Nikkhoo , C. H. Cheng , J. L. Wang , et al., “Development and Validation of a Geometrically Personalized Finite Element Model of the Lower Ligamentous Cervical Spine for Clinical Applications,” Computers in Biology and Medicine 109 (2019): 22–32, 10.1016/j.compbiomed.2019.04.010. 31035068

[54] A. Rohlmann , S. Neller , L. Claes , G. Bergmann , and H. J. Wilke , “Influence of a Follower Load on Intradiscal Pressure and Intersegmental Rotation of the Lumbar Spine,” Spine 26, no. 24 (2001): E557–E561, 10.1097/00007632-200112150-00014. 11740371

[55] D. C. Wilson , C. A. Niosi , Q. A. Zhu , T. R. Oxland , and D. R. Wilson , “Accuracy and Repeatability of a New Method for Measuring Facet Loads in the Lumbar Spine,” Journal of Biomechanics 39, no. 2 (2006): 348–353, 10.1016/j.jbiomech.2004.12.011. 16321637

[56] P. Brinckmann and H. Grootenboer , “Change of Disc Height, Radial Disc Bulge, and Intradiscal Pressure From Discectomy an In Vitro Investigation on Human Lumbar Discs,” Spine 16, no. 6 (1991): 641–646, 10.1097/00007632-199106000-00008. 1862403

[57] D. J. Polga , B. P. Beaubien , P. M. Kallemeier , et al., “Measurement of In Vivo Intradiscal Pressure in Healthy Thoracic Intervertebral Discs,” Spine 29, no. 12 (2004): 1320–1324, 10.1097/01.BRS.0000127179.13271.78. 15187632

[58] D. J. Pearsall , J. G. Reid , and L. A. Livingston , “Segmental Inertial Parameters of the Human Trunk as Determined From Computed Tomography,” Annals of Biomedical Engineering 24 (1996): 198–210, 10.1007/BF02667349. 8678352

[59] J. Clin , C. É. Aubin , N. Lalonde , S. Parent , and H. Labelle , “A New Method to Include the Gravitational Forces in a Finite Element Model of the Scoliotic Spine,” Medical & Biological Engineering & Computing 49 (2011): 967–977, 10.1007/s11517-011-0793-4. 21728065

[60] M. Dreischarf , T. Zander , G. Bergmann , and A. Rohlmann , “A Non‐Optimized Follower Load Path May Cause Considerable Intervertebral Rotations,” Journal of Biomechanics 43, no. 13 (2010): 2625–2628, 10.1016/j.jbiomech.2010.05.033. 20541208

[61] A. Rohlmann , L. Bauer , T. Zander , G. Bergmann , and H. J. Wilke , “Determination of Trunk Muscle Forces for Flexion and Extension by Using a Validated Finite Element Model of the Lumbar Spine and Measured In Vivo Data,” Journal of Biomechanics 39, no. 6 (2006): 981–989, 10.1016/j.jbiomech.2005.02.019. 16549091

[62] U. M. Ayturk and C. M. Puttlitz , “Parametric Convergence Sensitivity and Validation of a Finite Element Model of the Human Lumbar Spine,” Computer Methods in Biomechanics and Biomedical Engineering 14, no. 8 (2011): 695–705, 10.1080/10255842.2010.493517. 21229413

[63] A. Meiring , E. P. de Kater , A. Stadhouder , B. J. van Royen , P. Breedveld , and T. H. Smit , “Current Models to Understand the Onset and Progression of Scoliotic Deformities in Adolescent Idiopathic Scoliosis: A Systematic Review,” Spine Deformity 11, no. 3 (2023): 545–558, 10.1007/s43390-022-00618-1. 36454530

[64] S. Schmid , K. A. Burkhart , B. T. Allaire , D. Grindle , and D. E. Anderson , “Musculoskeletal Full‐Body Models Including a Detailed Thoracolumbar Spine for Children and Adolescents Aged 6–18 Years,” Journal of Biomechanics 102 (2020): 109305, 10.1016/j.jbiomech.2019.07.049. 31471110 PMC7315467

[65] S. Jia , L. Lin , H. Yang , J. Fan , S. Zhang , and L. Han , “The Influence of the Rib Cage on the Static and Dynamic Stability Responses of the Scoliotic Spine,” Scientific Reports 10, no. 1 (2020): 16916, 10.1038/s41598-020-73881-9. 33037307 PMC7547652

[66] R. Allais , A. Capart , A. da Silva , and O. Boiron , “Biomechanical Consequences of the Intervertebral Disc Centre of Rotation Kinematics During Lateral Bending and Axial Rotation,” Scientific Reports 13, no. 1 (2023): 3172, 10.1038/s41598-023-29551-7. 36823433 PMC9950088

[67] J. A. Wheeldon , F. A. Pintar , S. Knowles , and N. Yoganandan , “Experimental Flexion/Extension Data Corridors for Validation of Finite Element Models of the Young, Normal Cervical Spine,” Journal of Biomechanics 39, no. 2 (2006): 375–380, 10.1016/j.jbiomech.2004.11.014. 16321642

[68] I. Zafarparandeh , D. U. Erbulut , I. Lazoglu , and A. F. Ozer , “Development of a Finite Element Model of the Human Cervical Spine,” Turkish Neurosurgery 24, no. 3 (2014): 312–318, 10.5137/1019-5149.jtn.8486-13.0. 24848166

[69] D. J. Woldtvedt , W. Womack , B. C. Gadomski , D. Schuldt , and C. M. Puttlitz , “Finite Element Lumbar Spine Facet Contact Parameter Predictions Are Affected by the Cartilage Thickness Distribution and Initial Joint Gap Size,” Journal of Biomechanical Engineering 133 (2011): 061009, 10.1115/1.4004287. 21744929

[70] N. Yoganandan , S. Kumaresan , and F. A. Pintar , “Biomechanics of the Cervical Spine Part 2. Cervical Spine Soft Tissue Responses and Biomechanical Modeling,” Clinical Biomechanics 16, no. 1 (2001): 1–27, 10.1016/S0268-0033(00)00074-7. 11114440

[71] S.‐J. Tang , R. C. Dong , X. Cheng , Y. T. Liu , Z. L. Wang , and P. B. Zhang , “Effect of Anteroposterior Vibration Frequency on the Risk of Lumbar Injury in Seated Individuals,” Ergonomics 68, no. 7 (2025): 1067–1079, 10.1080/00140139.2024.2391591. 39150052

[72] R. Dong , S. Zhu , X. Cheng , X. Gao , Z. L. Wang , and Y. Wang , “Study on the Biodynamic Characteristics and Internal Vibration Behaviors of a Seated Human Body Under Biomechanical Characteristics,” Biomechanics and Modeling in Mechanobiology 23, no. 5 (2024): 1449–1468, 10.1007/s10237-024-01849-z. 38671153

[73] M. A. Adams and P. Dolan , “Spine Biomechanics,” Journal of Biomechanics 38, no. 10 (2005): 1972–1983, 10.1016/j.jbiomech.2005.03.028. 15936025

