# Supplementary material for: Development and Assessment of a Novel Generic Finite Element Spine Model for Clinical Applications
Source: Int J Numer Method Biomed Eng. 2025 Sep 22;41(9):e70098. doi: 10.1002/cnm.70098 (PMC12452808; doi:10.1002/cnm.70098)
Supplement: Supplementary file 1 — Data S1: Supporting Information. [file CNM-41-e70098-s001.docx]

**Development and assessment of a novel generic finite element spine model for clinical applications**

Yifan Su^1^ ([y.su-24@sms.ed.ac.uk](mailto:y.su-24@sms.ed.ac.uk)), Athanasios I. Tsirikos^2^ ([atsirikos@hotmail.com](mailto:atsirikos@hotmail.com)), Vasileios Koutsos^3^ ([vasileios.koutsos@ed.ac.uk](mailto:vasileios.koutsos@ed.ac.uk)), Pankaj Pankaj^1*^ ([pankaj@ed.ac.uk](mailto:pankaj@ed.ac.uk))

1. Institute for Bioengineering, School of Engineering, The University of Edinburgh, UK

2. Scottish National Spine Deformity Centre – Royal Hospital for Children and Young People, Edinburgh, UK

3. Institute for Materials and Processes, School of Engineering, The University of Edinburgh, UK

**Supplementary figures:**


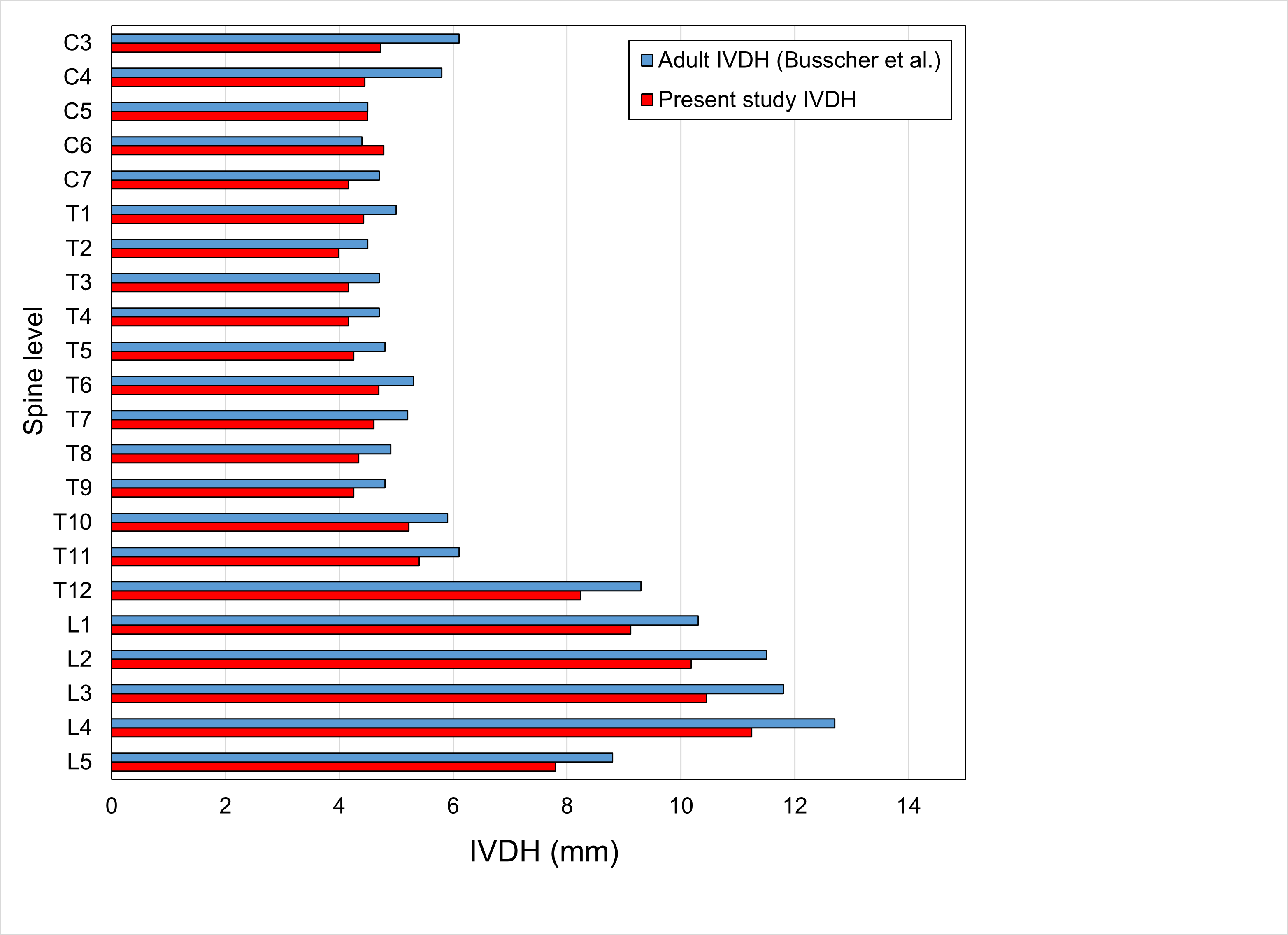


**Figure S1:** Sagittal vertical intervertebral disc height (IVDH) developed in this study compared to adult IVDH morphology from Busscher et al. [25]


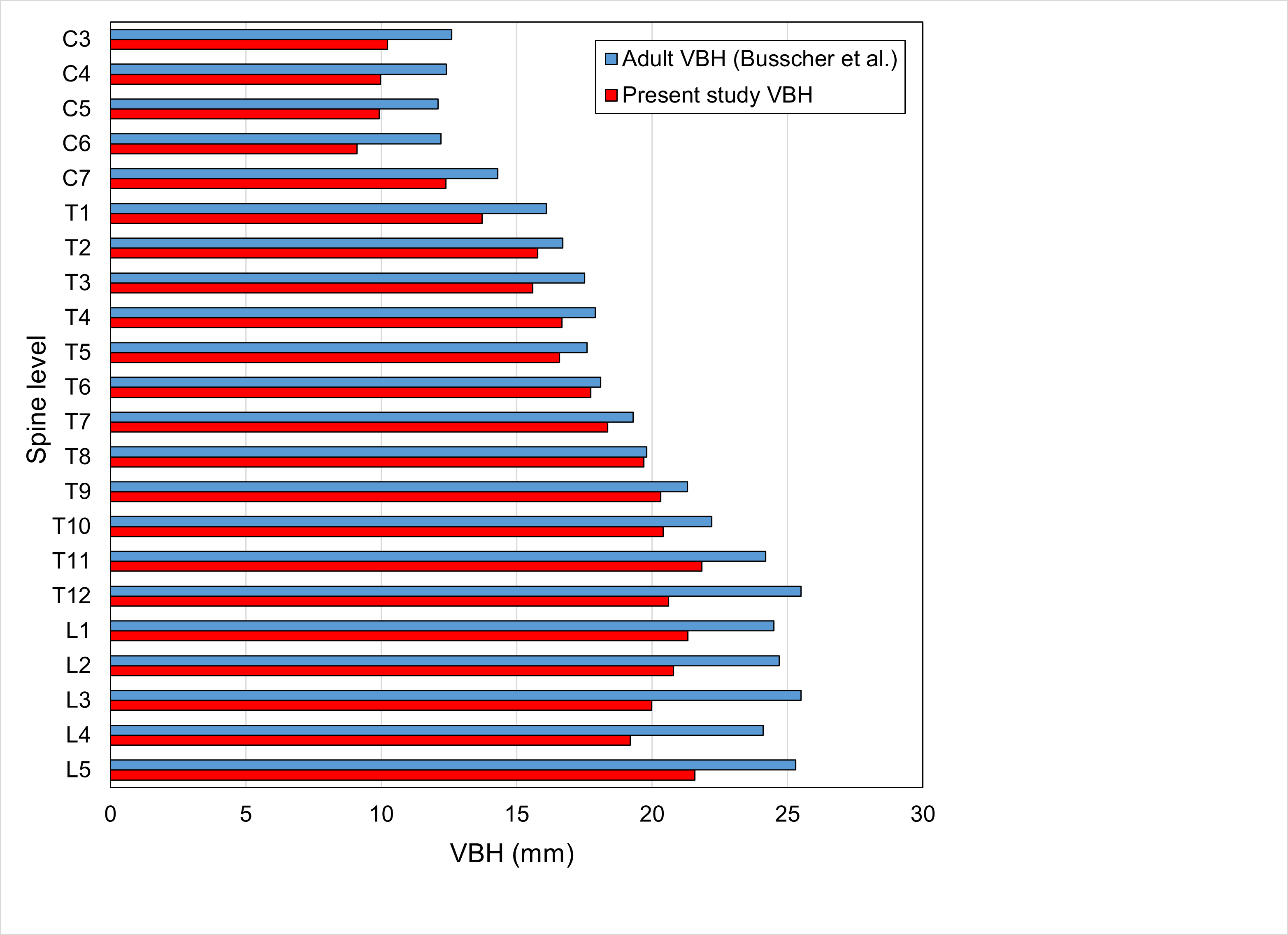


**Figure S2:** Sagittal vertical vertebral body height (VBH) of the generic model developed compared to adult VBH morphology from Busscher et al. [25]


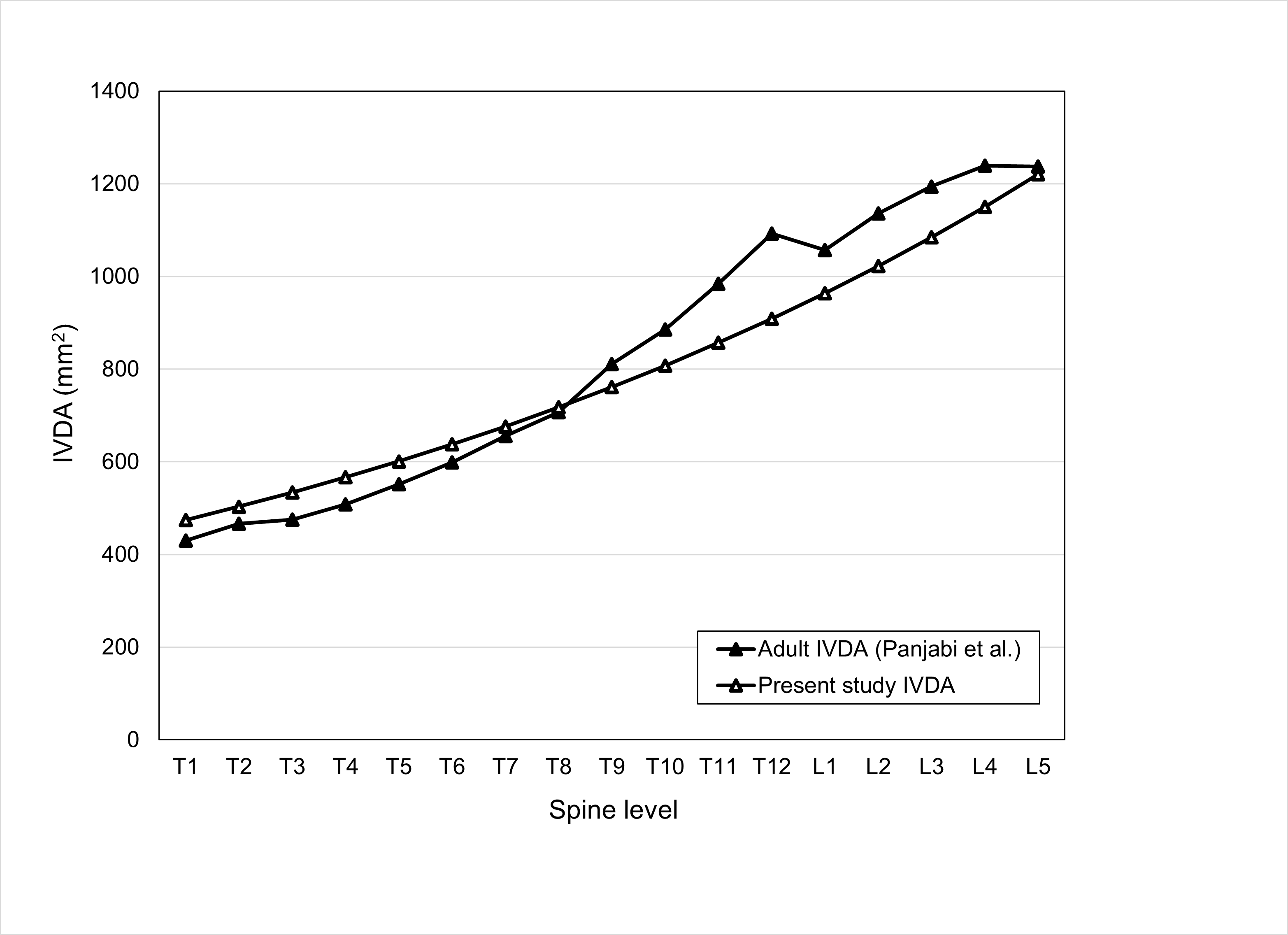


**Figure S3:** Intervertebral disc area (IVDA) of the generic model developed in this study compared to adult IVDA morphology from Panjabi et al.[23]


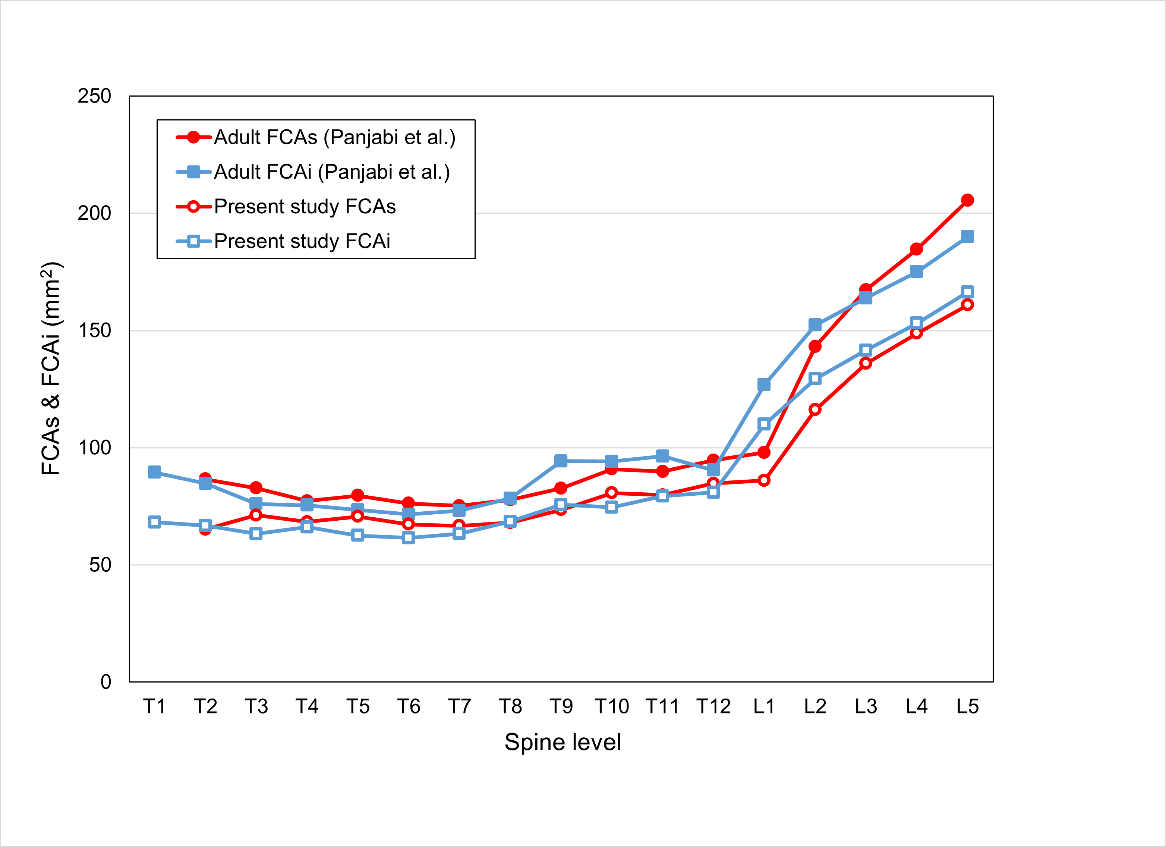


**Figure S4:** Superior facet area (FCAs) and inferior facet area (FCAi) of the generic model developed compared to adult FCAs and FCAi morphology from Panjabi et al. [1, 23]


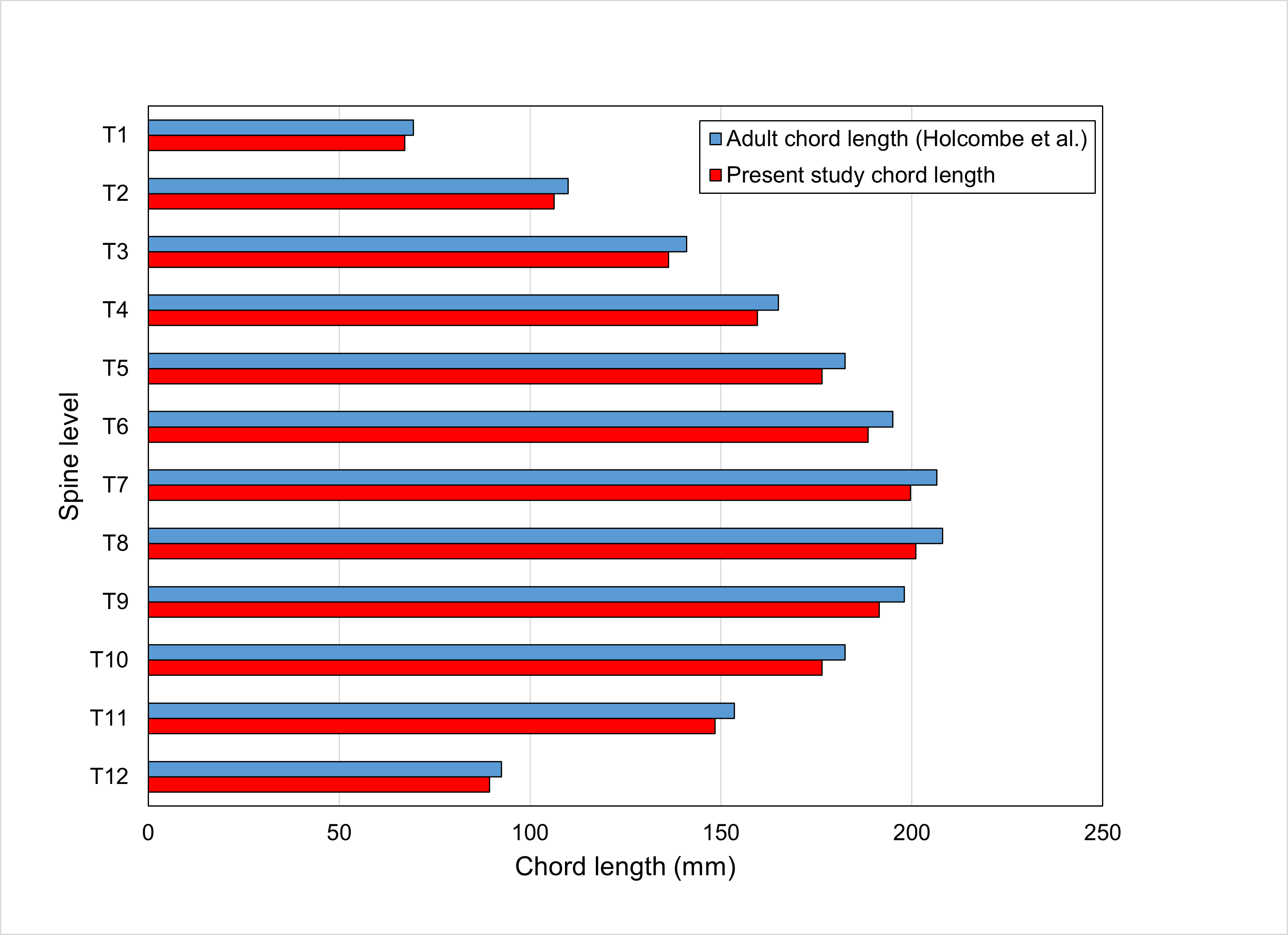


**Figure S5:** Chord length of the present ribcage model compared to adult chord lengths measured *in vitro* by Holcombe et al. [26]


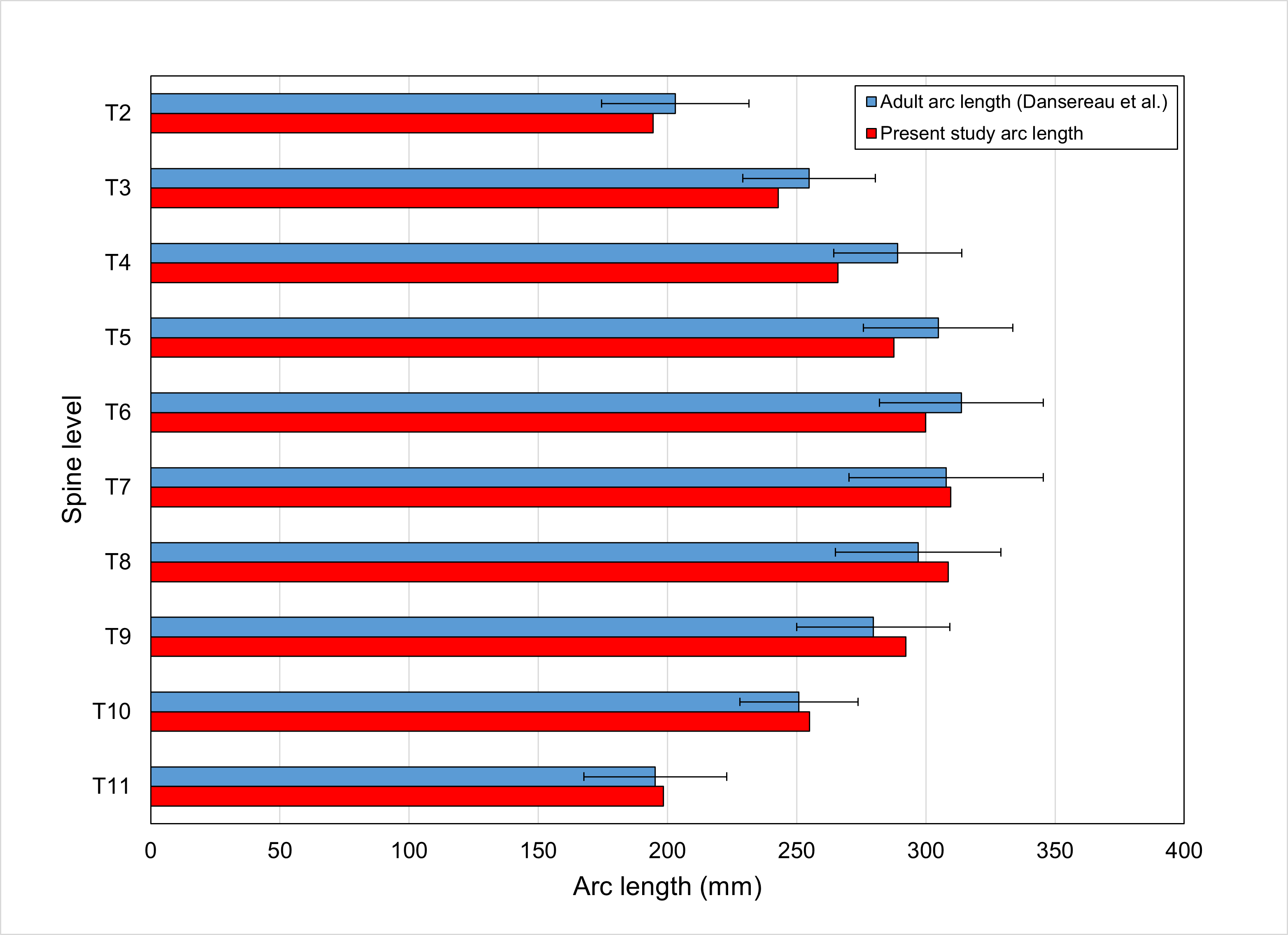


**Figure S6:** Arc length of the present ribcage model compared to adult arc lengths measured *in vitro* by Dansereau et al. [34]

**
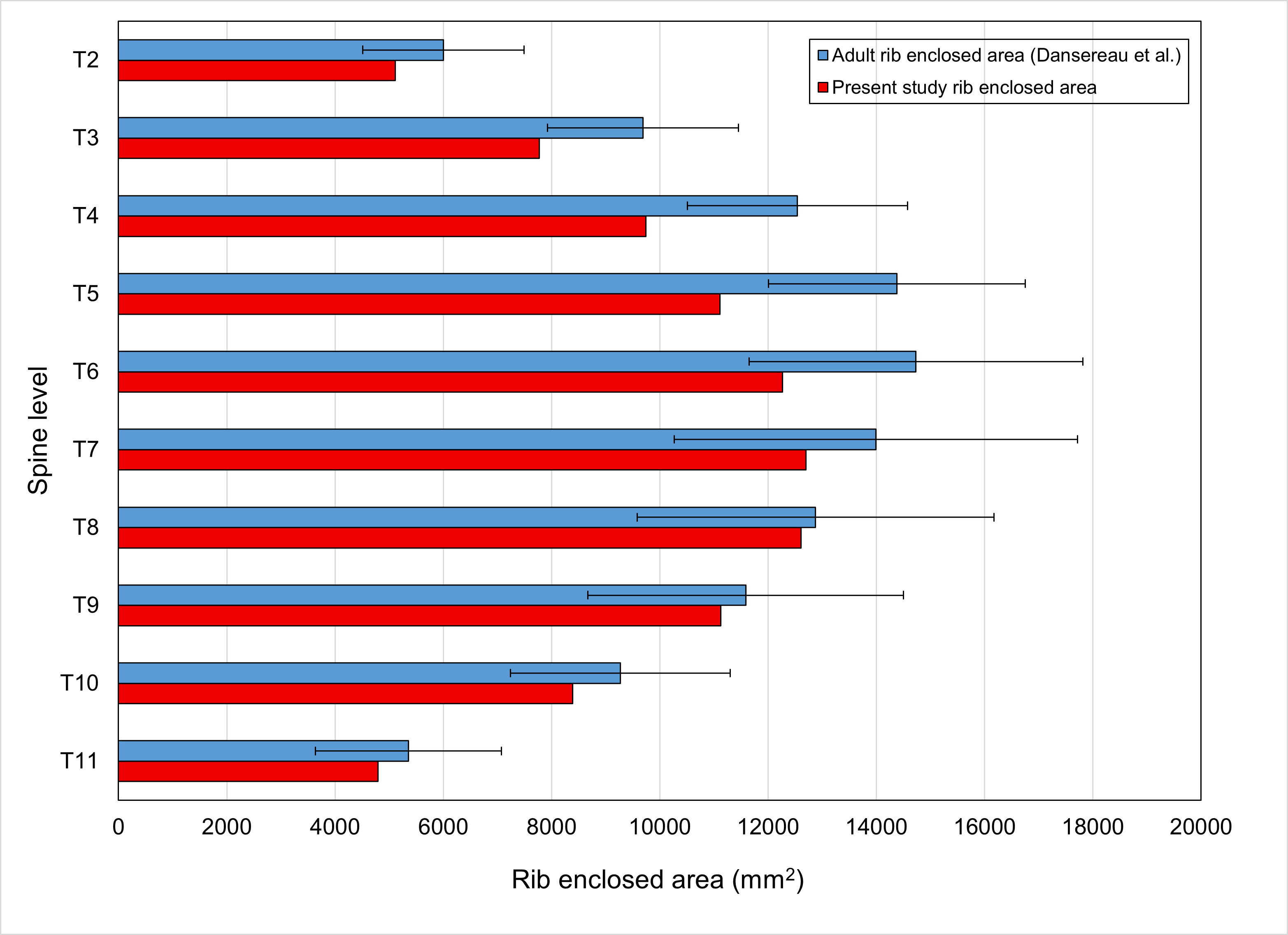
**

**Figure S7:** Enclosed area by the present rib model compared to adult rib enclosed area measured *in vitro* by Dansereau et al. [34]

**Supplementary tables:**

| **Table S1. Parameters used for developing the ribcage** | | | | | |  |  |
| --- | --- | --- | --- | --- | --- | --- | --- |
| Dimension | Proximal inner angle (º) | Chord length (mm) | Arc length (mm) | θ^1^ (º) | θ^2^ (º) | θ^3^ (º) | D (mm) |
| T1 | 80.5 | 67.19 | 113.73 | 52.33 | 38.37 | 27.63 | 183.37 |
| T2 | 112 | 106.34 | 194.48 | 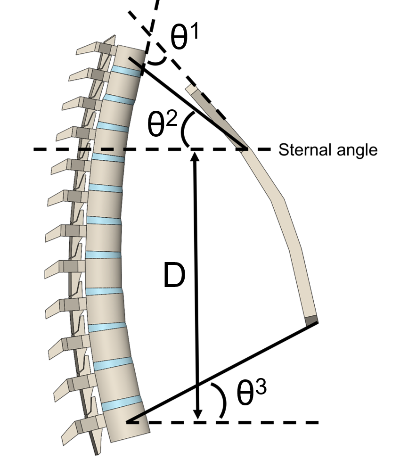 | | | |
| T3 | 116.5 | 136.31 | 242.84 |  |  |  |  |
| T4 | 115 | 159.51 | 266.02 |  |  |  |  |
| T5 | 113 | 176.43 | 287.63 |  |  |  |  |
| T6 | 112 | 188.51 | 299.83 |  |  |  |  |
| T7 | 109 | 199.63 | 309.62 |  |  |  |  |
| T8 | 104 | 201.08 | 308.66 |  |  |  |  |
| T9 | 98 | 191.41 | 292.25 |  |  |  |  |
| T10 | 90 | 176.43 | 255.04 |  |  |  |  |
| T11 | 82.5 | 148.39 | 198.46 |  |  |  |  |
| T12 | 60 | 89.42 | 113.53 |  |  |  |  |

| **Table S2. Mesh sensitivity analysis based on FJF results using five mesh schemes** | | | | | |
| --- | --- | --- | --- | --- | --- |
| Mesh scheme | 1.5 mm | 2 mm | 3 mm | 4 mm | 5 mm |
| Element counts | 420807 | 187682 | 58904 | 28013 | 15860 |
| Node counts | 636632 | 298314 | 105725 | 50041 | 30613 |
| Predicted FJF (N) | 105.551 | 105.519 | 105.879 | 105.754 | 106.88 |
| Percentage change compared to 1.5 mm mesh scheme | ⸺ | 0.03% | 0.31% | 0.19% | 1.26% |
| Percentage change between mesh schemes | 0.03% | 0.34% | 0.12% | 1.05% | ⸺ |

| **Table S3. Mesh sensitivity analysis based on IDP results using five mesh schemes** | | | | | |
| --- | --- | --- | --- | --- | --- |
| Mesh scheme | 1.5 mm | 2 mm | 3 mm | 4 mm | 5 mm |
| Element counts | 420807 | 187682 | 58904 | 28013 | 15860 |
| Node counts | 636632 | 298314 | 105725 | 50041 | 30613 |
| Predicted IDP (MPa) | 0.599 | 0.598 | 0.596 | 0.455 | 0.387 |
| Percentage change compared to 1.5 mm mesh scheme | ⸺ | 0.05% | 0.45% | 23.94% | 35.43% |
| Percentage change between mesh schemes | 0.05% | 0.40% | 30.87% | 17.78% | ⸺ |
